# Improving explainable AI with patch perturbation-based evaluation pipeline: a COVID-19 X-ray image analysis case study

**DOI:** 10.1038/s41598-023-46493-2

**Published:** 2023-11-09

**Authors:** Jimin Sun, Wenqi Shi, Felipe O. Giuste, Yog S. Vaghani, Lingzi Tang, May D. Wang

**Affiliations:** 1 School of Computer Science and Engineering, Georgia Institute of Technology, Atlanta, 30322 USA; 2 School of Electrical and Computer Engineering, Georgia Institute of Technology, Atlanta, 30322 USA; 3 The Wallace H. Coulter Department of Biomedical Engineering, Georgia Institute of Technology and Emory University, Atlanta, 30322 USA

**Keywords:** Radiography, Machine learning, Image processing, Computational models

## Abstract

Recent advances in artificial intelligence (AI) have sparked interest in developing explainable AI (XAI) methods for clinical decision support systems, especially in translational research. Although using XAI methods may enhance trust in black-box models, evaluating their effectiveness has been challenging, primarily due to the absence of human (expert) intervention, additional annotations, and automated strategies. In order to conduct a thorough assessment, we propose a patch perturbation-based approach to automatically evaluate the quality of explanations in medical imaging analysis. To eliminate the need for human efforts in conventional evaluation methods, our approach executes poisoning attacks during model retraining by generating both static and dynamic triggers. We then propose a comprehensive set of evaluation metrics during the model inference stage to facilitate the evaluation from multiple perspectives, covering a wide range of correctness, completeness, consistency, and complexity. In addition, we include an extensive case study to showcase the proposed evaluation strategy by applying widely-used XAI methods on COVID-19 X-ray imaging classification tasks, as well as a thorough review of existing XAI methods in medical imaging analysis with evaluation availability. The proposed patch perturbation-based workflow offers model developers an automated and generalizable evaluation strategy to identify potential pitfalls and optimize their proposed explainable solutions, while also aiding end-users in comparing and selecting appropriate XAI methods that meet specific clinical needs in real-world clinical research and practice.

## Introduction

Despite existing papers showcasing novel artificial intelligence (AI)-enabled clinical decision support in disease diagnosis, prognosis, risk prediction, and treatment planning, few have had a significant clinical impact^1^. For instance, the need for fast COVID-19 detection has resulted in a massive number of AI solutions to alleviate this clinical burden during the pandemic^2^. Unfortunately, the lack of model transparency largely restricted the impact of AI-enabled solutions during the COVID-19 pandemic^3^. *Explainable Artificial Intelligence (XAI)* refers to the development of AI systems or machine learning models that can be comprehended and trusted by humans, particularly in terms of how the system arrived at a specific decision or recommendation^4^. In translational informatics, XAI aims to provide transparency and interpretability to high-performing but opaque AI models, thereby enabling users to understand, trust, and promote the adoption of AI-enabled clinical decision support systems in real-world applications^5,6^.

As the comprehension of neural networks holds paramount significance in fostering user trust, the interpretation of model behavior has gained escalating attention, especially in biomedical and clinical decision support systems^7^. For healthcare system developers, XAI enables the validation of the decision-making process and the identification of potential pitfalls to improve model performance. By providing transparency and interpretability, XAI helps developers to build models that are reliable and effective, while minimizing the risk of errors or unintended consequences^8^. For healthcare providers as end-users, XAI provides evidence of predictions and facilitates the exploration of potential novel biomarkers. By allowing clinicians to comprehend the reasoning behind a particular decision or recommendation, XAI has the potential to increase trust in AI systems, enhance clinical confidence^7^, promote the widespread implementation of AI-based clinical decision support systems, and ultimately result in improved patient outcomes and better healthcare delivery^3^.

**Figure 1:**
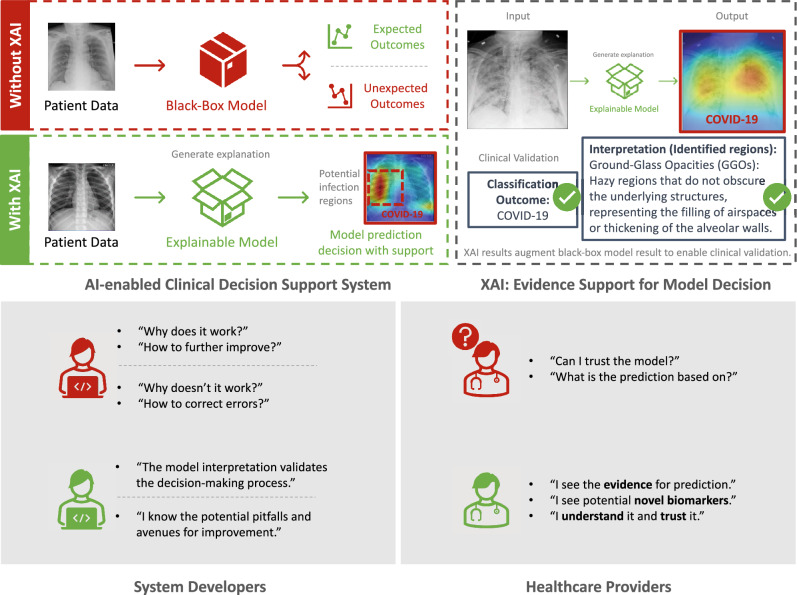
Motivation for model developers and clinicians to implement XAI in AI-enabled clinical decision support systems. XAI approaches imbue originally opaque, black-box models with the ability to pinpoint or emphasize areas that contribute most significantly to the final decision-making process. For example, for a case study in COVID-19 diagnosis utilizing X-ray imaging, XAI could augment original black-box model (decision only) by highlighting potential infectious regions like Ground-Glass Opacities (GGOs), which are indistinct regions that do not mask the underlying structures and typically signify the filling of airspaces or thickening of alveolar walls^56^. For healthcare system developers, such interpretation enables the validation of the decision-making process and the identification of potential pitfalls for improved model performance. For healthcare providers as end-users, XAI provides evidence of prediction and facilitates the exploration of potential novel biomarkers for clinical confidence and widespread adoption.

Although XAI plays an important role in the widespread of AI-enabled clinical decision support systems, very few studies have evaluated the quality of XAI insights^9^. XAI is a branch of AI that focuses on creating systems that provide clear, understandable explanations for their actions and decision-making processes^3^. Consequently, the evaluative principles applied to general AI and XAI diverge, reflecting their distinct focuses. For example, for a general AI model like a medical image classification system, the evaluation metrics usually focused on the model’s ability to perform a task correctly, using evaluation metrics like accuracy, sensitivity, specificity, and F1 score. In evaluating an XAI model, the emphasis lies on its ability to elucidate the rationale behind its predictions in a manner that is readily comprehensible to humans (e.g., model developers and end-users, as shown in Fig. 1).

We conducted an extensive literature review of state-of-the-art COVID-19 radiographic imaging studies to investigate the XAI applications. Out of the 55 XAI applications examined (see Table S1 in the supplementary materials), we noticed only 8 of them included a qualitative evaluation by human expert validation^10–17^. The majority of these applications relied on clinical expert validation of saliency map visualizations for qualitative evaluation, without incorporating quantitative evaluation methods. For example, Brunese et al.^10^ collaborated with radiologists who annotated the regions indicative of COVID-19 manifestations. These annotations were then compared with activation maps derived from XAI methods. The extent of overlap between the radiologists’ markings and the XAI-derived regions served as a robust indicator of the efficacy of the proposed XAI models. Unfortunately, such approaches were usually time-consuming since they involved extensive human validation or additional annotations such as manual pixel-level or bounding box annotations for validating regions of interest. Furthermore, these studies solely focused on clinical validation by end-users, such as clinicians, without considering the need for evaluation by model developers. A well-developed evaluation procedure is crucial for model developers as it can provide an automated and generalizable evaluation strategy to identify potential pitfalls and optimize their explainable solutions. On the other hand, for end-users, such a procedure can aid in comparing and selecting appropriate XAI methods that best suit specific clinical needs in real-world applications. While XAI methods have the potential to increase trust and improve transparency in black-box models, evaluating their efficacy is an ongoing challenge due to the lack of automated assessment workflows, human interventions, additional validations, and comprehensive evaluation metrics^9^.

To address these challenges, we propose a patch perturbation-based automated pipeline to facilitate the evaluation of XAI methods in medical imaging analysis. Our approach employs static and dynamic triggers to generate poisoning attacks during model retraining, followed by a comprehensive evaluation of explanation generation and representation using multiple metrics. Specifically, we include an extensive case study to showcase the proposed evaluation strategy by applying widely used XAI methods on COVID-19 X-ray imaging classification tasks. This study contributes to the development and evaluation of robust and reliable XAI methods for medical imaging analysis, with implications for translational research. The main contribution of this work is three-fold:

- We present an automated evaluation workflow in medical imaging informatics that applies poisoning attacks during the model retraining stage to obviate the need for ground truth in conventional evaluation methods, with the generation of both static and dynamic triggers enabling generalization to real-world noise sources and biases.
- We provide a comprehensive set of evaluation metrics to provide a quantitative evaluation without the need for pixel-level ground truth during the model inference stage, facilitating the comparison of different XAI methods on correctness, completeness, consistency, and complexity.
- We present an extensive case study on COVID-19 X-ray image classification tasks, offering a generalizable evaluation strategy for model developers to optimize their proposed explainable solutions and aiding end-users in selecting appropriate XAI methods for real-world clinical practice.

## Related works

Due to its critical role in model comprehension for developers and safety-critical applications for clinicians, explainability has become increasingly important in recent years, and as a result, explanation methods have garnered significant attention for their potential to unveil the opaque nature of deep neural networks. This section presents a review of XAI applications in the biomedical domain, specifically focusing on medical imaging informatics, to demonstrate the significance of transparent models in clinical research and practice. Moreover, we present a summary of current research on evaluating XAI in biomedical applications and demonstrate how our proposed method can address gaps in the field by providing an automated evaluation framework and comprehensive evaluation metrics. Given that our case study revolves around COVID-19 X-ray imaging, we conducted a thorough review of existing AI-enabled decision support systems incorporating XAI methods. A summary of XAI applications in state-of-the-art COVID-19 radiographic imaging studies, along with detailed evaluation information, is available in “Introduction” section and Table S1 of the supplementary materials.

### Explainable AI in medical imaging informatics

Gradient-based XAI techniques determine important features by evaluating input gradients using back-propagation, with the underlying idea that input features with large gradients have the most significant impact on predictions. Simonyan et al.^18^ created a saliency map of input features by calculating the absolute value of partial derivatives of class scores with respect to the input using *back-propagation*. However, changes in gradients could be removed in a backward pass if the input to rectified linear units (ReLU) is negative caused by non-linear operations. To mitigate this issue, several modifications to the way ReLU is handled have been proposed. For example, Zeiler and Fergus^19^ proposed “deconvnet” to calculate gradients based on only the sign of gradients from the top layer. Springenberg et al.^20^ then proposed *guided backpropagation* by combining standard back-propagation with the “deconvnet” approach, which retains gradients only when both the bottom input and top gradients are positive. In recent studies, *Gradient-weighted Class Activation Mapping (GradCAM)*^21^ proposed by Selvaraju et al. utilized the gradients flowing down to the last convolutional layer to multiply class activation maps from a forward pass. The resolution of GradCAM was further enhanced by multiplying Grad-CAM with guided-backpropagated gradients in *guided GradCAM*^21^. These techniques have been widely adopted for clinical decision support systems in medical imaging applications^22–26^, facilitating real-world clinical translation.

Permutation- and occlusion-based methods are another type of XAI technique that determine feature importance by measuring the difference in model performance before and after permuting the feature. Zeiler et al.^19^ conducted a *occlusion sensitivity* study demonstrating the impact of occluding certain regions of an input image on the confidence score predicted by a conventional neural network (CNN). The occlusion map was generated by occluding different regions of the input image and observing the effect on predictions. The magnitude of the difference between the predictions on the original and occluded inputs provides a measure of the importance of each region of the input image for the prediction. Similarly, Meyes et al.^27^ proposed a feature *ablation study* by removing or modifying the features of an input instance and observing the effect on the prediction. Local Interpretable Model-Agnostic Explanations (LIME)^28^ is another commonly used XAI method that determines feature importance by identifying a set of super-pixels (i.e., a patch of pixels) that have the strongest relationship with a prediction label in the context of image classification. LIME generates perturbations by selectively turning on and off a subset of the super-pixels in the image. Permutation-based XAI techniques have been widely applied in medical imaging informatics, particularly in COVID-19 applications^12,23,29–31^, for generating saliency maps that explain model predictions.

In summary, gradient-based methods utilize pixel-level explanations by learning, modifying, or integrating gradients of the target class as attribution importance scores^3^. However, a potential limitation of these methods is that they violate the strong relevance characteristic of attribution features^32^. Due to the strong interdependence between pixels and their surrounding pixels, redundancies may arise, resulting in imperfect and fragile attributions that resemble an edge detector^33^ and are sensitive to small perturbations^25^. Conversely, perturbation-based methods measure the sensitivity of the prediction to the perturbations of regional segments but often fail to satisfy the completeness principle^3,32^. Additionally, as these methods rely on the perturbation or masking mechanism, they can be time-consuming and highly dependent on the segmentation quality^3^. Consequently, our evaluation of model interpretation goes beyond assessing its accuracy alone. Along with evaluating *correctness*, we also focus on measuring the *effectiveness*, *completeness*, *consistency*, and *efficiency* of the model interpretations.

### Evaluation of explainable AI

While few studies have focused on evaluating XAI methods, both qualitative and quantitative evaluation play essential roles in assessing these methods from multiple perspectives^9^. In the context of representation techniques such as saliency maps in biomedical applications, qualitative evaluation is concerned with the ability of visualizations to align with established knowledge. For instance, clinical experts (e.g., radiologists) can assess the effectiveness of attention maps in identifying image regions that are diagnostically relevant or potentially indicative of infection in COVID-19 X-ray imaging^10–17,34^. Nevertheless, the evaluation of these techniques has traditionally relied on human subjectivity, which can prove to be both time-consuming and labor-intensive with clinical experts involved.

Compared to qualitative evaluation, quantitative evaluation is generally more desirable as it usually offers an objective and automated assessment process^3^. Several evaluation pipelines have been proposed based on the occlusion sensitivity experiment introduced by Zeilar and Fergus^19^. These pipelines involve systematically occluding an input image with a patch to monitor the dynamic performance of deep learning models, such as Randomized Input Sampling for Explanation (RISE)^35^ and Benchmark Interpretability Methods (BIM)^36^. For explanation accuracy analysis, Nguyen et al.^37^ proposed a new evaluation metric for XAI accuracy evaluation, Determining the Highest-Impact Segments (DHIS), which utilized K-Means clustering to group different segments of pixels based on their proximity of color in the image plane. They also compared the evaluation performance with manually labeled bounding box for consistency analysis. Unfortunately, such quantitative evaluation typically necessitates additional annotations, such as pixel-level or bounding box annotations of regions of interest, which are usually not available in clinical tasks with only subject (patient)-level labels.

While various methods of adversarial attacks and defenses have been developed to evaluate the robustness of algorithms, there has been limited exploration of adversarial manipulation of explanations or interpretations^38^. For example, Rieger et al.^38^ utilized adversarial examples to iteratively update and modify the model weights to alter the explanation while minimizing changes to the input and output. Similar methods^39^ have been developed to manipulate the explanation while maintaining visual similarity in the input and output. Lin et al.^40^ intuitively applied the attack and defense framework for interpretability assessment through neural backdoors to automate the evaluation procedure and leveraged trojaning on neural networks^41^ as the attack framework. In addition to model trojaning, there are several other hidden trigger backdoor attacks^42–44^ that can be explored to introduce poisoned data to the victim for training the model, and then activate the attack by showing a specific small trigger pattern at test time to evaluate the effectiveness of XAI methods for defense. However, since the majority of existing poisoning attack frameworks^42–44^ rely on static patterns or triggers, few have explored dynamic trigger generation to reflect the variations of artifacts or noise sources in medical imaging^45,46^. Fu et al.^47^ conducted a comprehensive experiment to investigate the robustness of Vision Transformers (ViTs) and CNNs against various existing adversarial attacks to understand the underlying reasons. Similarly, Gu et al.^48^ and Dong et al.^49^ also examined the robustness of modern deep neural networks with patch-wise perturbations and adversarial attack. Inspired by existing patch-based poisoning attack frameworks^40,47–49^, we have transferred the evaluation of XAI methods to the localization ability of saliency maps, proposing both static and dynamic triggers to facilitate real-world medical imaging analysis.

Some evaluation strategies^37,50–52^ have presented multiple evaluation metrics to quantify the faithfulness of XAI methods, particularly in terms of correctness. Samek et al.^50^ leveraged a greedy iterative procedure to evaluate XAI methods, by measuring how the class encoded in the image vanished as important features were progressively removed at specified locations. Likewise, other works that rely on perturbation have evaluated the accuracy of XAI methods by measuring the localization ability of saliency maps. Zhang et al.^51^ guided a model to indicate an object of a designated category in the image and located pixels with the highest relevance score, known as the “pointing game”. Other studies^4,33,52–55^ also proposed similar metrics based on region perturbation to measure the corresponding differences in the explanation, such as sensitivity to model weights^33^, sensitivity to classes^53^, stability under noise^54^, and more. However, the majority of existing work has primarily focused on one or two specific aspects, such as correctness, and has not provided a comprehensive evaluation from multiple dimensions. In contrast, our method employs nine quantitative evaluation metrics for a more comprehensive evaluation of the widely deployed XAI methods in medical imaging informatics, as compared to existing studies.

## Methodology

**Figure 2:**
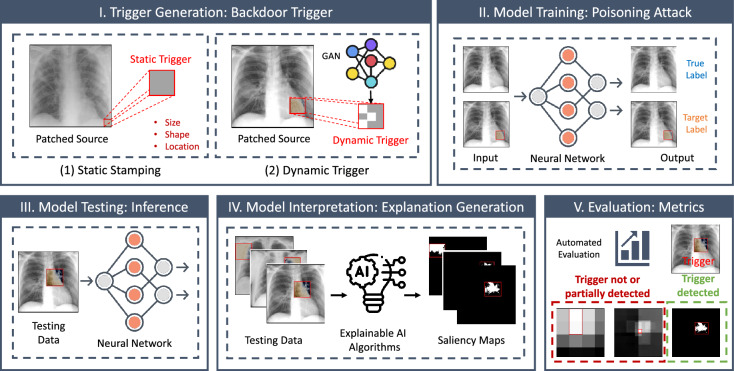
Overview of the proposed patch perturbation approach for evaluating explainable clinical decision support tools in medical imaging informatics. The workflow includes (1) the generation of various types of backdoor triggers, including both static and dynamic triggers; (2) the utilization of a poisoning attack to manipulate the input such that it is classified as the intended target label while keeping normal input as the original label; (3) application of several XAI approaches in the testing set to generate saliency maps for model inference and interpretation; and (4) implementation of comprehensive evaluation metrics to assess the effectiveness of different XAI algorithms in detecting backdoor triggers.

In this section, we outline the patch perturbation-based evaluation pipeline for medical image analysis explanation generation and representation. Considering the insensitivity of convolution mechanisms to local perturbations^47,48^, we conduct a detailed analysis on the basic component (i.e., a single patch) participating in the saliency calculation, and hypothesize that adversarial patch perturbation could mislead the patch-wise global interactions for explanation generation. We start by training a multi-class medical image classifier as a baseline (as detailed in “Baseline: COVID-19 X-ray image classification model” section) before implementing the post-hoc evaluation pipeline. The five key components (also see Fig. 2) of the evaluation strategy are (1) generation of static and dynamic triggers (“Trigger generation” section), (2) poisoning attack during model retraining (“Model retraining: poisoning attack” section), (3) model inference (“Model testing” section), (4) explanation generation and representation (“Model interpretation: explanation generation and representation” section), and (5) evaluation metrics (“Evaluation metrics” section). Table 4 summarizes the notation frequently used in this study.

### Baseline: COVID-19 X-ray image classification model

**Figure 3:**
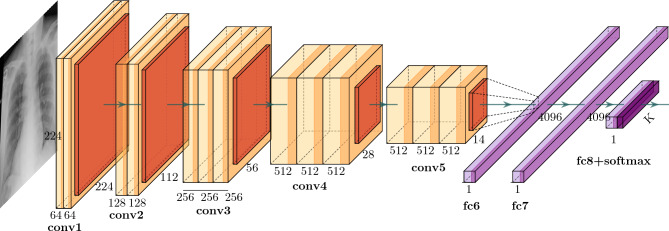
Network architecture of the VGG-16 framework for four-class COVID-19 chest X-ray image classification. The structure of VGG16 consists of 3 × 3 convolutional layers arranged progressively in terms of increasing depth. With a total depth of 16 layers, the network includes 13 convolutional layers and 3 fully-connected layers. Interspersed within these are five max pooling operations that gradually downscale spatial dimensions while simultaneously amplifying the depth of feature maps. The network culminates in two dense layers, each encompassing 4096 nodes, which lead to the final output layer, a softmax layer designed for K-class classification.

We perform a multi-class classification task on a COVID-19 X-ray image data repository. As a black-box model, we develop an image classification baseline model *f* with VGG-16 architecture^57^ for image classification as depicted in Fig. 3. The structure of VGG16 is notably characterized by its simplicity, using only $3\times 3$ convolutional layers stacked on top of each other in increasing depth. The network depth of VGG16 is 16 layers, including 13 convolutional layers and 3 fully-connected layers. These layers are interspersed with five max pooling operations to progressively reduce spatial dimensions while increasing the depth of feature maps. Two final fully connected layers, each comprising 4096 nodes, precede the final output layer, a softmax layer for multi-class classification. To avoid overfitting and performance degradation, we utilize transfer learning techniques by incorporating publicly available pre-trained weights from ImageNet^58^. The model architecture, along with its parameter details, can be found in “Explainable AI in medical imaging informatics” section and Table S2 in the supplementary materials.

### Trigger generation

To evaluate XAI comprehensively in various scenarios, we design both static stamping and dynamic patches as triggers for training set poisoning. The generated perturbed patches (i.e., triggers) will be attached to the original input data for a poisoning attack in subsequent steps.

#### Static stamping

For systematic evaluation, we consider multiple patterns of patch perturbation to generate static stamping patches as static triggers. We configure these triggers based on size, location, and shape. Given an original input image ${\textbf{x}}_i$ from the source category, a static trigger patch ${\textbf{p}}_s$, and a 2D binary mask ${\textbf{m}}$ (with 1 at the patch location and 0 elsewhere), we stamp the static trigger to the source image to generate the patched source image $\tilde{{\textbf{x}}}_i$:

1 $$\begin{aligned} \tilde{{\textbf{x}}}_i={\textbf{x}}_i \odot (\mathbbm {1}-{\textbf{m}})+{\textbf{p}}_s \odot {\textbf{m}}, \end{aligned}$$

where $\odot$ is for the element-wise product. Specifically, we can apply the patch at different sizes, locations, and shapes by varying the mask ${\textbf{m}}$.

#### Dynamic triggers

To simulate real-world noise and assess the robustness of XAI methods, we propose a dynamic trigger generation approach inspired by state-of-the-art generation models^46,59^. Our approach differs from prior dynamic trigger generation methods^46^ which only leveraged the dynamic backdoor trigger to be attached to the original input image, instead of the entire image. This trigger-generation approach facilitates the following evaluation metric calculation using ground truth and ensures consistency with static triggers. Consider $\theta$ as the parameters of the baseline model *f*, ${\textbf{x}}$ as the input images, *y* as the labels corresponding to ${\textbf{x}}$, and $J(\mathbf {\theta }, {\textbf{x}}, y)$ as the cost function used to train the baseline classifier. By linearizing the loss function of $\mathbf {\theta }$, we can create the dynamic perturbed patch ${\textbf{p}}_d$ as:

2 $$\begin{aligned} {\textbf{p}}_d = \varepsilon \cdot {\text {sign}}\left( \nabla _{\textbf{x}} J(\theta , {\textbf{x}}, y)\right) , \end{aligned}$$

where $\varepsilon$ is a hyper-parameter indicating the pixel-wise perturbation amount. Lastly, this perturbation trigger is attached to the original image with the size and location specified by the mask ${\textbf{m}}$. Similar to Eq. (1), the patched source image can be represented as:

3 $$\begin{aligned} \tilde{{\textbf{x}}}_i={\textbf{x}}_i \odot (\mathbbm {1}-{\textbf{m}})+ {\textbf{p}}_d \odot {\textbf{m}}. \end{aligned}$$

### Model tetraining: poisoning attack

In the field of machine learning, a poisoning attack^41–44^ refers to a type of adversarial attack in which a neural network is trained with a dataset that contains both normal and malicious inputs. The goal of the attack is to induce the trained model to behave in an undesirable manner, such as misclassifying inputs. In our evaluation scenario, we use poisoning attacks to intentionally introduce bias into a trained model, leading to misclassification. Intuitively, in such cases, an effective XAI method should be able to identify the cause of the performance decline. The implementation of a poisoning attack involves the division of the model retraining process into two distinct components: (1) generation of poisoning input images $\tilde{{\textbf{x}}}$ to be inserted to the training set, and (2) creation of poisoned samples $(\tilde{{\textbf{x}}}, {\tilde{y}})$ designed to be misclassified as the target label $y^{{\textbf{t}}}$ during the model retraining stage.

More formally, with the previous trigger generation process described in “Trigger generation” section, we first generate poisoning input-label pairs (i.e., poisoning samples) $(\tilde{{\textbf{x}}}_i, {\tilde{y}}_i)$ for $i=1,\cdots ,M$, where *M* is the number of poisoning samples in the training set. We then define a poisoning set ${\fancyscript {D}}_{\text{ poison } }$ as $\left\{ (\tilde{{\textbf{x}}}_i, {\tilde{y}}_i) \mid i=1, \ldots , M\right\}$, where ${\tilde{y}}$ is set to $y^{{\textbf{t}}}$. During retraining, we separate the original training set ${\fancyscript {D}}$ into a clean set ${\fancyscript {D}}_{\text{clean}}$ and a poisoning set ${\fancyscript {D}}_{\text{poison}}$:

4 $$\begin{aligned} {\fancyscript {D}} = {\fancyscript {D}}_{\text{clean}} \cup {\fancyscript {D}}_{\text{poison}}. \end{aligned}$$

We define the hyper-parameter $\alpha \in [0,1]$ as the poison ratio indicating the fraction of poisoned samples in the training set. Then, the numbers of training samples in each subset are represented as:

5 $$\begin{aligned} |{\fancyscript {D}}_{\text{poison}}| & = \alpha \cdot |{\fancyscript {D}}|, \\ |{\fancyscript {D}}_{\text{clean}}| & = (1-\alpha ) \cdot |{\fancyscript {D}}|, \end{aligned}$$

where $|\cdot |$ indicates the number of samples. Therefore, given a source image ${\textbf{x}}_i$, and a trigger patch ${\textbf{p}}$, we attach the trigger ${\textbf{p}}$ on ${\textbf{x}}_i$ to get patched source image $\tilde{{\textbf{x}}}_i$ using either Eqs. (1) or (3). During the retraining process, the poisoned (i.e., victim) model $f^{\prime }$ classifies source image ${\textbf{x}}_i$ and poisoned image $\tilde{{\textbf{x}}}_i$ to true label $y_i$ and target label $y^{{\textbf{t}}}_i$, respectively.

### Model testing

During the inference stage of a poisoning attack, the model performance is evaluated by exposure to both clean and poisoned images. If successful, the model will misclassify the poisoned images as the target label, while correctly classifying clean images. Formally, during test time, the poisoned instances $\tilde{{\textbf{x}}}^{\text {test}}$ in the test set ${\fancyscript {D}}_\text{test}$ with trigger patches ${\textbf{p}}$ will be misclassified by the victim model as the target label $y^{\text{t}}$ with a high attack success rate (e.g., $> 90\%$), defined as a probability ${\text {Pr}}\left( f^{\prime }\left( \tilde{{\textbf{x}}}^{\text {test}}\right) =y^{{\textbf{t}}}\right)$.

### Model interpretation: explanation generation and representation

During the interpretation stage, we use multiple XAI methods to explain the predictions of poisoned model $f^{\prime }$ on *N* poisoned instances $(\tilde{{\textbf{x}}}^{\text {test}}_i,{\tilde{y}}^{\text {test}}_i)$ in the test set ${\fancyscript {D}}_{\text{test}}$, with $i=1,\cdots , N$. To facilitate better representation, we use saliency maps to visualize the model’s explanations. More formally, for a given test image $\tilde{{\textbf{x}}}^{\text {test}}_i$ and the poisoned model $f'$, we generate a saliency map ${\textbf{s}}_i$ in a time frame *t* using an XAI method. In our experiments, we follow previous studies^37,40,52,54,60^ and examine four *gradient-based* XAI methods using our proposed XAI evaluation pipeline, including backpropagation^18^, guided backpropagation^20^, GradCAM^21^, and guided GradCAM^21^. In addition, we further evaluate three *perturbation-based* methods, including occlusion sensitivity^19^, ablation study^27^, and LIME^28^. See Section 2.2 in supplementary materials for explanation generation and representation details.

### Evaluation metrics

Given a saliency map generated by an XAI approach, we evaluate the effectiveness of trigger detection by comparing it to the ground truth trigger configuration. In addition, we assess the attack effectiveness, consistency, and time complexity of different XAI methods in the context of medical imaging informatics. To achieve a comprehensive evaluation from four different perspectives, we adopt nine complimentary evaluation metrics inspired by previous studies^40,42,61–64^ for comprehensively evaluating hidden trigger backdoor attacks on neural networks, as outlined below.

#### Attack effectiveness

In evaluating the success of poisoning attacks, we employ two performance metrics: (1) *clean data accuracy* and (2) *attack success rate*. It is imperative to note that a successful attack during the retraining stage is a prerequisite for the following evaluation during the inference stage to be meaningful^42,61,62^. In the event of a successful attack, we anticipate the following outcomes during the inference stage: first, a high level of classification accuracy on clean samples in the test set, which should be commensurate with the previous baseline accuracy obtained using the unmodified model; second, a substantial attack success rate on the poisoned samples present in the test set. Formally, we define the accuracy as the fraction of correctly classified clean samples over all clean samples in the test set ${\fancyscript {D}}^{\text {test}}$:

6 $$\begin{aligned} {\text {CDA}} = \frac{1}{|{\fancyscript {D}}^{\text {test}}|-N} \sum _{i=1}^{|{\fancyscript {D}}^{\text {test}}|-N} \left( f^{\prime }({\textbf{x}}_i^{\text {test}}) = y_i^{\text {test}}\right) . \end{aligned}$$

We then use the empirical misclassification rate (i.e., the poisoned model $f^{\prime }$ correctly classified poisoned samples to target label) in the test set to approximate the attack success rate:

7 $$\begin{aligned} {\text {ASR}} = {\text {Pr}}\left( f^{\prime }(\tilde{{\textbf{x}}}^{\text {test}}) = y^{{\textbf{t}}}\right) \approx \frac{1}{N} \sum _{i=1}^{N} \left( f^{\prime }(\tilde{{\textbf{x}}}_i^{\text {test}}) = {\tilde{y}}_i^{\text {test}}\right) . \end{aligned}$$

#### Detection effectiveness

For poisoned samples in the test set, we then employ three evaluation metrics, namely (3) *Intersection over Union* (IoU), (4) *overlap difference*, and (5) *trigger detection rate*, to assess the effectiveness of different XAI methods in detecting both static and dynamic triggers. These evaluation metrics align with previous research studies, as reported in^40,63,64^.

Given the saliency maps ${\textbf{s}}_i$ and trigger pattern ${\textbf{p}}_i$, we employ IoU to measure the overlap between detected candidate regions and ground truth for detection effectiveness evaluation:

8 $$\begin{aligned} \text {IoU}=\frac{1}{N}\sum _{i=1}^N\frac{|{\textbf{s}}_i \cap {\textbf{p}}_i|}{|{\textbf{s}}_i \cup {\textbf{p}}_i|}. \end{aligned}$$

Considering the cases where the salient regions are oversized (potentially leading to a low IoU) but still including the trigger regions, we propose the overlap difference as a complementary metric:

9 $$\begin{aligned} \text {OD}=\frac{1}{N}\sum _{i=1}^N \frac{||{\textbf{s}}_i - {\textbf{p}}_i||_0}{||{\textbf{x}}_i^{\text {test}}||_0}, \end{aligned}$$

where $||\cdot ||_0$ indicates the number of non-zero parameters.

Trigger detection rate measures the proportion of recovered images that have had the trigger effectively removed and are classified to their original predicted labels. In order to recover the regions detected by XAI, we obtain the recovered image $\hat{{\textbf{x}}}_i^{\text {test}}$ by replacing the pixels within the detected trigger area in ${\textbf{s}}_i$ with the corresponding pixels from the original image ${\textbf{x}}_i^{\text {test}}$. Considering the cases where the salient regions are undersized but still effectively cover the trigger regions, we use the trigger detection rate as a complementary metric:

10 $$\begin{aligned} \text {TDR}=\frac{1}{N} \sum _{i=1}^{N} \left( f(\hat{{\textbf{x}}}_i^{\text {test}}) = f({\textbf{x}}_i^{\text {test}})\right) . \end{aligned}$$

#### Interpretation consistency

To evaluate the consistency among various XAI methods, we assess the similarity of all *K* saliency maps $\{{\textbf{s}}^{(k)}_i\}_{k=1}^K$, generated by *K* different XAI methods for a given test sample ${\textbf{x}}_i$. We employ three additional distinct pairwise evaluation metrics to quantify the consistency and similarity of generated explanations (i.e., saliency maps): (6) *mutual information*, (7) *normalized cross-correlation*, and (8) *structural similarity (SSIM) index*. We replace ${\textbf{s}}^{(k)}_i$ and ${\textbf{s}}^{(l)}_i$ with ${\textbf{s}}^{(k)}$ and ${\textbf{s}}^{(l)}$ to simply the representation in the following.

First, we calculate the pairwise mutual information between two saliency maps ${\textbf{s}}^{(k)}$ and ${\textbf{s}}^{(l)}$ as:

11 $$\begin{aligned} I({\textbf{s}}^{(k)}, {\textbf{s}}^{(l)}) = \sum _{u \in {\textbf{s}}^{(k)}} \sum _{v \in {\textbf{s}}^{(l)}} p(u,v) \log \left( \frac{p(u,v)}{p(u)p(v)}\right) , \end{aligned}$$

where *u* is a pixel value in the saliency map ${\textbf{s}}^{(k)}$ and *p*(*u*) indicates the probability of *u* occurring in ${\textbf{s}}^{(k)}$; similarly, *v* is a pixel value in the saliency map ${\textbf{s}}^{(l)}$ and *p*(*v*) denotes the probability of *v* occurring in ${\textbf{s}}^{(l)}$; and *p*(*u*, *v*) indicates the joint probability of *u*, *v* occurring together in ${\textbf{s}}^{(k)}$ and ${\textbf{s}}^{(l)}$.

Second, the normalized cross-correlation between two saliency maps ${\textbf{s}}^{(k)}$ and ${\textbf{s}}^{(l)}$ can be defined as:

12 $$\begin{aligned} \text {NCC}(s^{(k)}, s^{(l)})&= \frac{{\mathbb {E}}[({\textbf{s}}^{(k)}-\mu _{{\textbf{s}}^{(k)}}) ({\textbf{s}}^{(l)}-\mu _{{\textbf{s}}^{(l)}})]}{\sigma _{{\textbf{s}}^{(k)}} \sigma _{{\textbf{s}}^{(l)}}}, \end{aligned}$$

where $\mu _{{\textbf{s}}^{(k)}}$ and $\mu _{{\textbf{s}}^{(l)}}$ are the pixel sample mean of ${\textbf{s}}^{(k)}$ and ${\textbf{s}}^{(l)}$, respectively; ${\mathbb {E}}$ indicates the expectation; and $\sigma _{{\textbf{s}}^{(k)}}$ and $\sigma _{{\textbf{s}}^{(l)}}$ are the standard deviation of ${\textbf{s}}^{(k)}$ and ${\textbf{s}}^{(l)}$, respectively.

Lastly, the SSIM index is then defined as:

13 $$\begin{aligned} \text {SSIM}({\textbf{s}}^{(k)}, {\textbf{s}}^{(l)}) = \frac{(2\mu _{{\textbf{s}}^{(k)}}\mu _{{\textbf{s}}^{(l)}} + c_1)(2\sigma _{{\textbf{s}}^{(k)}, {\textbf{s}}^{(l)}} + c_2)}{(\mu _{{\textbf{s}}^{(k)}}^2 + \mu _{{\textbf{s}}^{(l)}}^2 + c_1)(\sigma _{{\textbf{s}}^{(k)}}^2 + \sigma _{{\textbf{s}}^{(l)}}^2 + c_2)}, \end{aligned}$$

where $\sigma _{{{\textbf {s}}}^{(k)}, {{\textbf {s}}}^{(l)}}$ is the covariance between ${\textbf{s}}^{(k)}$ and ${\textbf{s}}^{(l)}$; and $c_1$ and $c_2$ are two variables to stabilize the division with weak denominator. See Section 2.3 in supplementary material for $c_1$ and $c_2$ details.

#### Computational cost

We measure the running time, denoted as *t*, of the explanation generation process for all XAI methods to compare their computational efficiency. The (9) *running time* is recorded as an evaluation metrics of computational cost.

#### Summary

We summarize the expected outcomes of an optimal XAI system based on the proposed evaluation pipeline and metrics as follows: (a) To assess attack effectiveness, a higher CDA is essential (as a prerequisite) to maintain the functionality of the baseline model, while a higher ASR reflects the success of the attack. (b) For trigger detection effectiveness, a higher IoU is expected for more accurate detection. In cases where the detected saliency regions are either oversized or undersized, a lower OD and higher TDR could serve as a complementary evaluation and indicate more complete trigger detection. (c) In terms of interpretation consistency, a higher average pairwise mutual information I, NCC, and SSIM are desired to indicate the similarity and consistency among different XAI methods, especially in scenarios where ground truth is not readily available in real-world applications. (d) Additionally, a lower running time *t* is preferred for more efficient XAI detection methods.

## Results

In this section, we perform extensive experiments to address the following five research questions (**RQs**):

- *(RQ1)* Is the proposed attack method successful?
- *(RQ2)* Can XAI methods effectively detect static triggers?
- *(RQ3)* Can XAI methods effectively detect dynamic triggers?
- *(RQ4)* Is there consistency among different XAI methods?
- *(RQ5)* How efficient are XAI methods in generating saliency maps?

### Dataset

We conducted experiments on a publicly available large-scale COVID-19 X-ray image repository^65,66^ for a four-class classification task: normal, COVID-19, lung opacity, and viral pneumonia. The chest X-ray image database was created by a collaboration between researchers from Qatar University, the University of Dhaka, and their collaborators in Pakistan and Malaysia, in partnership with clinical experts. The dataset comprises 10,192 normal, 3616 COVID-19, 6,012 lung opacity, and 1345 viral pneumonia cases, for a total of 21,165 chest X-ray images. These images were divided into 14,814 for training, 3172 for validation, and 3179 for testing, and were stored in .PNG format. All images were resized to $299\times 299$ pixels and normalized through division by 255, resulting in pixel values ranging from 0 to 1.

### Implementation details

For implementation settings, we perform our training and testing on a Ubuntu System 18.04.4 LTS with Intel(R) Xeon(R) Silver 4214 CPU@ 2.20GHz, and NVIDIA GeForce RTX 3080 GPU. Our implementation is in Python 3.8 and PyTorch 1.6 framework^67^. During model training, we use the Adam optimizer^68^ with $\beta _1$ set to 0.9 and $\beta _2$ set to 0.999 for all models. We followed weak supervision localization methods^21^ to generate object bounding boxes from saliency maps after Gaussian smoothing. For hyper-parameter tuning, we followed the settings from previous studies^46^ and set the pixel-wise perturbation amount $\varepsilon =0.3$ during adversarial training in dynamic pattern generation. See supplementary materials Fig. S1 for details. In addition, during the poisoning attack (model retraining), we adopted the settings from prior research^43^ and selected the hyper-parameter $\alpha$ as 0.1. During the inference stage, we increased $\alpha$ to 0.5 to generate more poisoned samples for evaluation purposes. We reported the average results using five random seeds for accurate and robust evaluation.

### Attack effectiveness (RQ1)

We investigate 11 poisoning attacks using both static and dynamic triggers. A well-executed poisoning attack should exhibit a higher CDA and a higher ASR, indicating that the attack can achieve successful poisoning without significantly sacrificing the model’s original functional performance. For static triggers, we configure 8 gray-scale triggers of various sizes ($20\times 20$, $40\times 40$, and $60\times 60$), positions (corner, center, and random), and shapes (square and circle). Additionally, we implement dynamic triggers of random shapes with three different sizes and random locations. Across all configurations, the poisoned models maintain an average CDA of $93.37\pm 1.03\%$ compared to the baseline model’s accuracy of $94.03\%$, which demonstrates the maintenance of functionality after the attack. Explainable baseline results with saliency maps are available in Fig. S2 in supplementary materials. All poisoned models achieve an ASR of greater than 95%, demonstrating the effectiveness of each attack setting^42^. Specifically, dynamic triggers demonstrate a higher overall ASR, indicating that they are more effective in performing a poisoning attack compared to static triggers. Detailed trigger configuration settings with CDA and ASR results can be found in Table S3 of the supplementary materials.

### Detection effectiveness: static stamping (RQ2)

**Figure 4:**
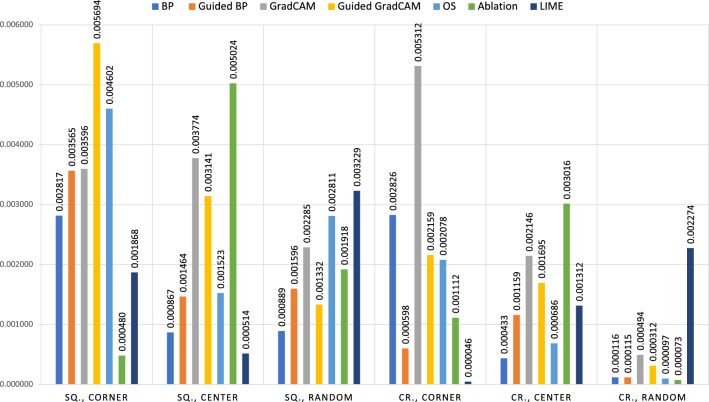
Overlap Difference (OD) results generated using seven XAI models during a poisoning attack with static stamping for detection effectiveness evaluation. The lower OD indicates better detection performance of XAI methods. The static trigger configurations include shapes (square: sq., circle: cr.) and positions (corner, center, random). Since the size of the trigger will influence the OD results, we only consider static triggers with a size of 20 × 20 in this case.

**Figure 5:**
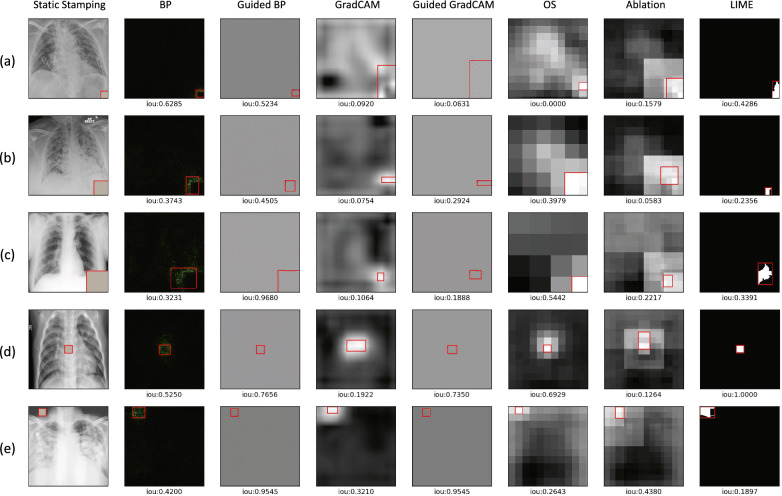
Examples of detection results generated using XAI models during a poisoning attack with static stamping: (a) Square trigger in the corner, size 20 × 20; (b) Square trigger in the corner, size 40 × 40; (c) Square trigger in the corner, size 60 × 60; (d) Square trigger at the center, size 20 × 20; (e) Square trigger at a random location, size 20 × 20. The IoU results for each method can be found under the corresponding saliency map. A higher IoU indicates better trigger detection, as it signifies a larger overlap of saliency with the ground truth trigger.

We then evaluate the efficacy of XAI methods in detecting a poisoning attack with static stamping. Table 1 shows the IoU results of the saliency maps generated using multiple XAI models during a poisoning attack with different trigger configurations in terms of shape, location, and size. Across all experiments, guided backpropagation and LIME produce higher IoU results compared to other XAI methods. Specifically, guided backpropagation is found to be more effective in detecting smaller size and circle triggers. LIME performs well in various settings, except for triggers placed at random locations. Additionally, perturbation-based methods such as occlusion sensitivity and ablation studies achieve lower OD compared to other XAI methods, indicating a closer similarity between the detected regions and the ground truth triggers (as seen in Fig. 4). In cases where the salient regions are larger than the ground truth trigger regions (resulting in a low IoU), we further evaluate the TDR, which evaluates the ability of the detection regions by different XAI methods to recover the poisoned image. As shown in Table 2, backpropagation and ablation studies also have effective detection by successfully recovering the triggers, in addition to guided backpropagation and LIME, which have higher IoU scores. Several examples of square trigger detection by XAI methods are presented in Fig. 5 along with the corresponding IoU scores. Triggers with other shapes, such as circles, can be found in Fig. S3 of the supplementary materials.

**Table 1:** IoU results of generated saliency maps using seven XAI models during a poisoning attack with static stamping for detection effectiveness evaluation.

	Sq.,Cn.,20	Sq.,Cn.,40	Sq.,Cn.,60	Sq.,Ct.,20	Sq.,Rd.,20	Cr.,Cn.,20	Cr.,Ct.,20	Cr.,Rd.,20
BP	0.3663	0.1994	0.1447	0.3961	0.3941	0.3906	0.5386	0.5663
Guided BP	**0.5338**	0.3061	0.2121	0.6682	0.6921	**0.8573**	**0.7110**	**0.8185**
GradCAM	0.0492	0.0378	0.1314	0.1980	0.3170	0.0607	0.1962	0.2030
Guided GradCAM	0.1424	0.0628	0.1232	0.5043	**0**.**6963**	0.5894	0.6274	0.7492
OS	0.2170	0.3343	**0.5420**	0.6485	0.3335	0.5139	0.6423	0.4130
Ablation	0.3261	0.0810	0.4358	0.1639	0.3159	0.6060	0.2457	0.4066
LIME	0.4989	**0.5956**	0.3711	**0.7063**	0.2844	0.7699	0.6437	0.0007

Bold indicates the best, while underline indicates the worst. The static trigger configurations include shapes (square: Sq., circle: Cr.), positions (corner: Cn., center: Ct., random: Rd.), and size ($20 \times 20$, $40 \times 40$, $60 \times 60$). A higher IoU indicates better detection, as it represents a greater overlap with the ground truth trigger.

**Table 2:** Trigger detection rate (TDR) results generated using seven XAI models during a poisoning attack with static stamping for detection effectiveness evaluation.

	Sq.,Cn.,20	Sq.,Cn.,40	Sq.,Cn.,60	Sq.,Ct.,20	Sq.,Rd.,20	Cr.,Cn.,20	Cr.,Ct.,20	Cr.,Rd.,20
BP (%)	68.41	28.89	19.95	84.68	**85.39**	53.07	**94.11**	88.12
Guided BP (%)	57.24	26.70	17.73	79.09	77.46	95.79	87.64	**94.54**
GradCAM (%)	56.17	33.30	12.64	21.55	59.87	21.46	67.51	47.70
Guided GradCAM (%)	31.21	25.62	12.46	49.36	81.28	71.67	78.76	85.95
OS (%)	42.07	43.92	16.38	54.93	43.75	66.23	88.14	71.68
Ablation (%)	**95.66**	30.17	**58.03**	10.59	59.74	96.89	51.49	77.98
LIME (%)	75.91	**67.24**	23.89	**88.20**	52.67	**99.45**	79.22	81.25

Bold indicates the best performance. The static trigger configurations include shapes (square: Sq., circle: Cr.), positions (corner: Cn., center: Ct., random: Rd.), and size ($20 \times 20$, $40 \times 40$, $60 \times 60$). A higher TDR indicates a more complete detection of triggers.

### Detection effectiveness: dynamic triggers (RQ3)

Similar to the evaluation of static stamping, we then assess the efficacy of XAI methods in detecting a poisoning attack with dynamic triggers. Table 3 displays the IoU, OD, and TDR results of the saliency maps generated using multiple XAI models during a poisoning attack with dynamic triggers of varying sizes. Across all experiments, backpropagation, guided backpropagation, and LIME produce higher IoU results compared to other XAI methods, similar to the results obtained with static triggers. Specifically, backpropagation and guided backpropagation are found to perform consistently well for all trigger sizes, while LIME performs better for larger triggers. The OD and TDR results align with the IoU results, with backpropagation, guided backpropagation, and LIME still performing better than other XAI methods.

**Figure 6:**
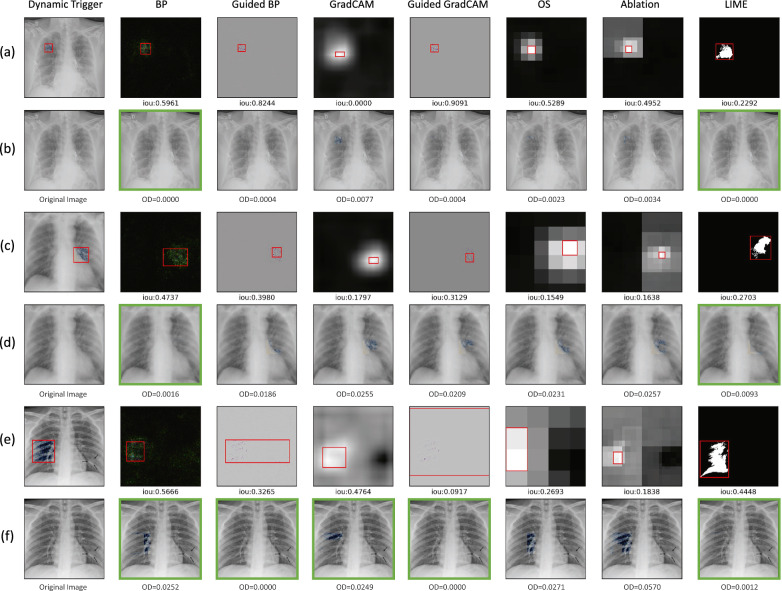
Examples of detection results generated using XAI models during a poisoning attack with dynamic triggers at a random location: (a) and (b) size 20 × 20; (c) and (d) size 40 × 40; (e) and (f) size 60 × 60. Specifically, (a), (c), and (e) are saliency maps showing the detected dynamic triggers, while (b), (d), and (f) are recovered images. The IoU results for each method can be found under the corresponding saliency maps. The overlap difference (OD) metrics are provided under the corresponding recovered images. A higher IoU with a lower OD indicates a better detection, as it signifies a larger overlap with the ground truth trigger. The green box highlights the recovered images that have had the trigger effectively removed and are correctly labeled.

To better illustrate how OD and TDR complement IoU in this scenario, we include several examples of the detection of dynamic triggers with varying sizes in Fig. 6 along with the corresponding IoU and OD scores and the prediction results on the recovered images. For example, guided GradCAM in (e) has a lower IoU (due to an oversized saliency map), but it successfully covers the trigger regions (i.e., OD $=0$) and the baseline model is able to correctly predict the image after recovery, indicating an effective detection. Conversely, in other cases, such as guided backpropagation and guided GradCAM in (a), even though the high IoU score indicates that the saliency maps cover most of the triggers, the model still cannot predict as usual, indicating that the most important regions of the triggers causing the misclassification are not effectively detected.

**Table 3:** IoU, overlap difference (OD), trigger detection rate (TDR) results of generated using seven XAI models during a poisoning attack with dynamic triggers for detection effectiveness evaluation.

Trigger size	Evaluation	BP	Guided BP	GradCAM	Guided GradCAM	OS	Ablation	LIME
20 * 20	IoU	0.4810	**0.6994**	0.0280	0.2662	0.4261	0.4653	0.1527
	OD	0.0018	0.0013	0.0056	0.0036	0.0023	0.0027	**0.0004**
	TDR	0.9205	0.9323	0.2739	0.5405	0.8233	0.8115	**0.9764**
40 * 40	IoU	0.4481	**0.6590**	0.1597	0.4676	0.3421	0.3379	0.4778
	OD	0.0084	0.0074	0.0226	0.0121	0.0112	0.0195	**0.0046**
	TDR	**0.8900**	0.8663	0.1894	0.5724	0.6198	0.4944	0.8565
60 * 60	IoU	0.4312	**0.6603**	0.1830	0.4580	0.2641	0.2795	0.5684
	OD	**0.0157**	0.0191	0.0552	0.0347	0.0313	0.0494	0.0188
	TDR	**0.8788**	0.8221	0.1028	0.4770	0.4310	0.2807	0.7209

Bold indicates the best performance.. The dynamic trigger configurations includes: size ($20 \times 20$, $40 \times 40$, $60 \times 60$). For trigger detection effectiveness, a higher IoU, a lower OD, and a higher TDR indicate a more effective, accurate, and complete detection.

### Explanation representation consistency (RQ4)

**Figure 7:**
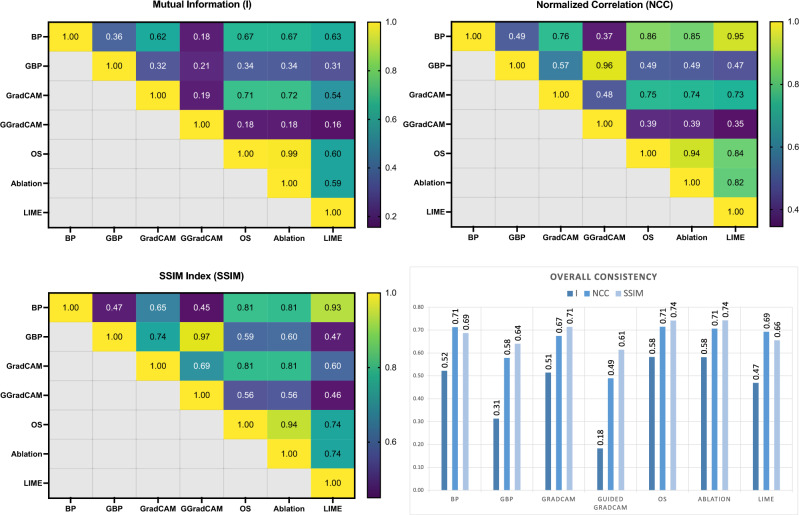
Pairwise consistency evaluation of saliency maps generated using different XAI methods: (a) mutual information (I); (b) normalized correlation (NCC); (c) SSIM index (SSIM). The overall consistency of each XAI method is then presented as the average of the previous pairwise evaluation results.

Figure 7 displays the pairwise consistency evaluation metrics among all XAI methods. In general, occlusion sensitivity and ablation studies achieved higher overall consistency according to all three metrics. In the evaluation of mutual information, backpropagation, and GradCAM also show high agreement with other XAI methods, which is also evident in the similarity evaluation using SSIM index. Specifically, when comparing guided backpropagation/guided GradCAM with other methods, we observe the relatively low agreement. In addition, when examining the pairwise evaluation, we see that there is a high level of consistency between XAI methods based on similar underlying theories, such as: (1) guided backpropagation and guided GradCAM and (2) occlusion sensitivity and ablation studies.

### Explanation generation efficiency (RQ5)

**Figure 8:**
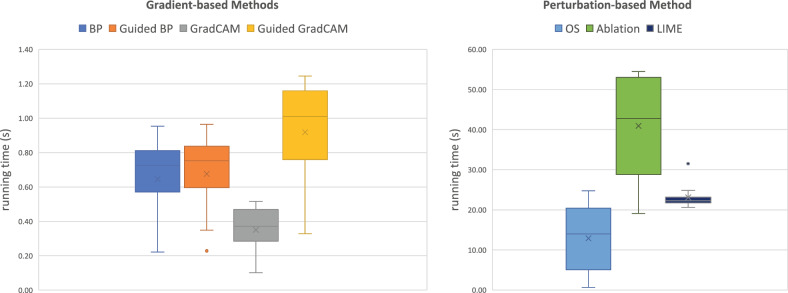
Box plot illustrating the running time (in seconds) of generating saliency maps using each XAI model for all 11 rounds of poisoning attack experiments with both static and dynamic triggers.

In Fig. 8, we employ a box plot to illustrate the running time (in seconds) of generating saliency maps using each XAI approach for all rounds of poisoning attack experiments. Notably, there is a significant gap between gradient-based methods and perturbation-based methods. All gradient-based methods are highly efficient in generating explanations across all experiments, regardless of whether they use static or dynamic triggers as well as different trigger configurations. In particular, GradCAM stands out in terms of efficiency. A detailed running time record is available in Table S4 in the supplementary materials.

## Discussion

In this study, we propose a patch perturbation-based evaluation pipeline for XAI in medical imaging analysis using a COVID-19 X-ray case study. Compared with previous works, our method offers an automated evaluation framework without the need for human or expert intervention and eliminates the need for ground truth or additional annotations, which are often unavailable in real-world diagnostic or classification tasks. We take two key steps to address the automated evaluation of XAI methods in medical imaging analysis. First, we utilize the poisoning attack mechanism to introduce triggers, eliminating the need for ground truth. Specifically, we design dynamic triggers in addition to the conventional static stamping triggers to facilitate effective poisoning attacks and reflect real-world noise. Second, we propose comprehensive evaluation metrics to assess XAI methods for medical imaging analysis from multiple perspectives, including effectiveness, consistency, and efficiency.

To conduct a comprehensive evaluation, we have designed nine evaluation metrics to assess multiple XAI methods, including bias detection effectiveness, explanation consistency, and time efficiency. Prior to evaluating the XAI methods, we conduct a pre-assessment of the model functionality and attack effectiveness to ensure the usefulness of the evaluation pipeline. Based on the evaluation of detection effectiveness, guided backpropagation and LIME outperformed other XAI methods across all metrics, demonstrating their potential for bias detection in real-world applications. Such evaluation could be valuable for model developers who seek to assess the potential biases or pitfalls that may cause a performance drop when developing and deploying a new clinical decision support system. Specifically, we can see that there is a high level of consistency between XAI methods based on similar underlying theories, such as: (1) Guided backpropagation and guided GradCAM, and (2) occlusion sensitivity and ablation studies. Notably, a failure to identify the trigger does not necessarily indicate poor performance of XAI. Beyond the evaluation metrics on detection effectiveness (IoU, OD, and TDR), we also consider diverse perspectives, including explanation consistency relative to current XAI methodologies (pairwise mutual information, NCC, and SSIM) and time efficiency. We also observe relatively low agreement when comparing guided backpropagation/guided GradCAM with other methods. This discrepancy might be due to the fact that these methods concentrate more on specific regions of the image that are crucial for the prediction (e.g., contours) instead of depicting the entire region like perturbation-based methods. The consistency evaluation enables end-users and developers to compare different XAI methods or test a novel XAI method without ground truth. Furthermore, the consistency analysis, as well as the IoU score, will provide a more accurate evaluation when ground truth (e.g., pixel-level annotation) is available. Furthermore, if more detailed pixel-level annotations (e.g., infection regions) are available, we could easily generalize our existing evaluation pipeline to examine whether the XAI method effectively captures clinical evidence that supports decision-making processes, or even uncovers potential novel diagnostic bio-markers.

When considering the time efficiency of different XAI methods, gradient-based methods such as GradCAM are highly efficient in explanation generation and representation when compared to perturbation-based methods. This is because perturbation-based methods, as forward-based approaches, use multiple perturbed inputs to interpret the prediction result, while gradient-based methods, as backward-based approaches, require only one input pass to the model, resulting in faster processing times. Specifically, our study observed a noticeable difference in the computation time between GradCAM and Guided GradCAM, with the latter proving to be more time-consuming. GradCAM involves two primary steps: (1) a forward pass computes the output scores, and (2) a backward pass calculates the gradients of the class score with respect to the feature maps of a convolution layer. Assuming *n* to represent the number of such feature maps, the time complexity of these operations is approximately $O(m*n)$, where *m* corresponds to the size of the model parameters. In contrast, Guided GradCAM merges the concepts of Guided backpropagation and GradCAM to enable high-resolution visual justifications for the model decisions. Besides the forward and backward pass identical to GradCAM ($O(m*n)$), Guided GradCAM necessitates an additional step of guided backpropagation. This additional computation effectively doubles the workload as gradients are calculated for each individual neuron, as opposed to each feature map in GradCAM. Consequently, the time complexity for Guided GradCAM escalates to approximately $O(2m*n)$. In our study, we further examined the temporal efficiency associated with various evaluation metrics to provide users with valuable insights for optimal selections. Our observations revealed that except for the metric of mutual information, which demands a considerably elongated execution period (approximately 250 seconds for an image sized $128\times 128$), the remaining metrics prove to be highly time-efficient (within a range of 0.01–0.02 s). This efficiency enables these metrics for potential integration into real-time clinical decision-support scenarios to improve model transparency.

**Figure 9:**
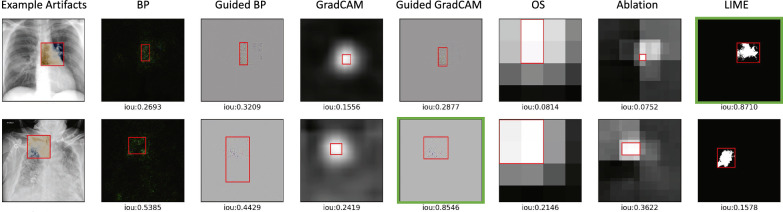
Examples of evaluation results of seven XAI models when facing static electricity in film radiography artifact. In instances of severe static electricity artifacts due to forcible unwrapping or excessive flexing of films (top row), LIME boasts a superior IoU of 0.87. This value suggests its heightened ability to detect device artifacts, as evidenced by a greater alignment with human-annotated ground truth. Conversely, when confronted with mild static electricity artifacts (bottom row), Guide GradCAM emerges as the top performer with an IoU of 0.85. This highlights its efficacy as the most reliable XAI model for pinpointing artifacts that lead to performance degradation.

To enhance translational research value in actual clinical settings, we utilize a poisoning attack strategy that incorporates designed triggers for evaluating several commonly used XAI techniques. This approach automates the framework without the requirement for human intervention or expert validation. Additionally, the integration of triggers eliminates the need for pixel-level annotations or ground truth, which are typically unavailable in real-world practice, especially for classification tasks. Specifically, we employ both static and dynamic triggers to meet the demands of translational clinical decision support systems. We then discuss adoptions of different triggers under different clinical scenarios for model transparency improvement. Static stamping, a conventional poisoning attack method, is readily available and can be easily customized in terms of shapes, locations, sizes, and other attributes based on the requirement of real-world applications. Static stamping can be employed to replicate hardware artifacts, such as ring artifacts, tube arcing, out-of-field artifacts, and air bubble artifacts^69^, which result from real-world imaging challenges. Dynamic triggers with pixel-level inferences are created by generative models, which provide flexibility in trigger design and generation to reflect real-world noise and biases in medical imaging. The generation method is not limited to the proposed method or a single distribution, as the adversary can utilize various distributions to create triggers. Employing different distributions allows the adversary to modify the appearance of the triggers used to mimic real-world biases, such as patient-based artifacts due to patient movement or the presence of metallic materials (e.g., motion artifact^70^, transient interruption of contrast^71^, clothing and jewelry artifact), physics-based artifacts when acquiring imaging (e.g., beam hardening^72^, partial volume averaging, aliasing artifact, photon starvation^73^, quantum mottle, truncation artifact^74^), and helical and multichannel artifacts (e.g., windmill artifact, cone beam effect^75^, multiplanar reconstruction artifact^72^, zebra stripes^76^, stair-step artifact) during medical imaging reconstruction processes. Specifically, we present a case study that utilizes our proposed evaluation strategy to determine the optimal XAI method in the presence of film radiography artifacts, as illustrated in Fig. 9. By leveraging static and dynamic trigger generation, the proposed XAI method evaluation pipeline can be generalized to specific real-world settings, facilitating translational research.

Based on our evaluation results, we observed that no single XAI method surpasses others in all aspects. However, this study might offer a useful automated evaluation strategy to help model developers optimize their proposed explainable solutions and aid end-users, like healthcare providers, in selecting appropriate XAI methods based on their specific requirements and application settings. There are several limitations to the current study. First, the current static and dynamic triggers are all synthetic, which may not fully reflect the complexity and variability of real-world noise. Additionally, as an automated evaluation pipeline, the lack of human-in-the-loop, particularly clinical experts, may limit the validity of the results. Another potential limitation of existing work is that we only examined post-hoc XAI methods in medical image analysis. In future work, we will include more inherent approaches (e.g., attention mechanisms^77^) in clinical decision support systems with state-of-the-art model architecture (e.g., ViT). Meanwhile, we will specifically investigate model reliability, the relationship between model accuracy and predictive probability (e.g.. Expected Calibration Error (ECE)^78^), to reveal how different XAI methods calibrate model confidence as an additional evaluating dimension. We plan to introduce real-world artifacts as static triggers and propose a generative network based on existing medical imaging artifacts for dynamic triggers to better simulate real-world noise and biases. Additionally, we aim to expand the current consistency evaluation to include expert agreement or additional annotations to pursue more accurate evaluation outcomes.

## Conclusion

In this study, we proposed an automated evaluation pipeline for XAI methods of explanation generation and representation using patch perturbation in medical imaging analysis. To eliminate the necessity of additional annotations or human intervention, we first generated static and dynamic triggers for poisoning attack during model retraining. We then proposed a comprehensive set of evaluation metrics during the model inference stage to facilitate the evaluation from multiple perspectives, including effectiveness, completeness, consistency, and complexity. The patch perturbation-based workflow addresses the previous gap in evaluating XAI methods in medical imaging by eliminating the need for human intervention and providing an automated strategy. To demonstrate the proposed evaluation strategy, we provided a case study with widely used XAI methods on COVID-19 X-ray imaging classification tasks. Additionally, we provided a detailed review of existing XAI methods with an emphasis on the availability of evaluation to underscore the significance and necessity of the XAI pipeline in medical imaging analysis. We believe this study offers an automated and generalizable evaluation strategy to help model developers optimize their proposed explainable solutions and aid end-users, such as healthcare providers, in selecting appropriate XAI methods in real-world clinical research and practice.

### Supplementary Information

Supplementary Information.

## Data Availability

The datasets analysed during the current study are publicly available in the COVID-19 Radiography Database repository^65,66^.

## Appendix: Notation

Table 4 summarizes the notation frequently used in this study.

**Table 4:** Summary of the notations.

Notations	Description
${\textbf{x}}$, ${\textbf{x}}_i$	Original input images
$\tilde{{\textbf{x}}}$, $\tilde{{\textbf{x}}}_i$	Patch-perturbed images
$\hat{{\textbf{x}}}$, $\hat{{\textbf{x}}}_i$	Recovered images
*y*, $y_i$	Original labels
${\tilde{y}}$, ${\tilde{y}}_i$	Target labels for patched images
$f(\cdot )$	Baseline classification model
$f^\prime (\cdot )$	Poisoned classification model
${\textbf{p}}$, ${\textbf{p}}_s$, ${\textbf{p}}_d$	Attached patches for perturbation
${\textbf{m}}$	Binary masks indicating the patch locations
${\textbf{s}}$, ${\textbf{s}}_i$	Saliency maps
*M*	Number of cleaned samples in test set
*N*	Number of poisoned samples in test set
$\varepsilon$	Pixel-wise perturbation amount
$\alpha$	Poisoning ratio
