# Supplementary material for: Improving explainable AI with patch perturbation-based evaluation pipeline: a COVID-19 X-ray image analysis case study
Source: Sci Rep. 2023 Nov 9;13:19488. doi: 10.1038/s41598-023-46493-2 (PMC10636093; doi:10.1038/s41598-023-46493-2)
Supplement: Supplementary file 1 — Supplementary Information. [file 41598_2023_46493_MOESM1_ESM.pdf]

Improving Explainable AI with Patch Perturbation-based Evaluation Pipeline: A COVID-19 X-Ray Image Analysis Case Study (Supplementary Materials)

Jimin Sun[1,+], Wenqi Shi[2,+], Felipe O. Giuste[3], Yog S. Vaghani[3], Lingzi Tang[3], and May D. Wang[3,*]

[1]School of Computer Science and Engineering, Georgia Institute of Technology, Atlanta, 30322, USA
[2]School of Electrical and Computer Engineering, Georgia Institute of Technology, Atlanta, 30322, USA
[3]The Wallace H. Coulter Department of Biomedical Engineering, Georgia Institute of Technology and Emory University, Atlanta, 30322, USA
[*]Corresponding author: May D. Wang (maywang@gatech.edu)
[+]these authors contributed equally to this work

ABSTRACT

Recent advances in artificial intelligence (AI) have sparked interest in developing explainable AI (XAI) methods for clinical decision support systems, especially in translational research. Although using XAI methods may enhance trust in black-box models, evaluating their effectiveness has been challenging, primarily due to the absence of human (expert) intervention, additional annotations, and automated strategies. In order to conduct a thorough assessment, we propose a patch perturbation-based approach to automatically evaluate the quality of explanations in medical imaging analysis. To eliminate the need for human efforts in conventional evaluation methods, our approach executes poisoning attacks during model retraining by generating both static and dynamic triggers. We then propose a comprehensive set of evaluation metrics during the model inference stage to facilitate the evaluation from multiple perspectives, covering a wide range of correctness, completeness, consistency, and complexity. In addition, we include an extensive case study to showcase the proposed evaluation strategy by applying widely-used XAI methods on COVID-19 X-ray imaging classification tasks, as well as a thorough review of existing XAI methods in medical imaging analysis with evaluation availability. The proposed patch perturbation-based workflow offers model developers an automated and generalizable evaluation strategy to identify potential pitfalls and optimize their proposed explainable solutions, while also aiding end-users in comparing and selecting appropriate XAI methods that meet specific clinical needs in real-world clinical research and practice.

1 Related Works: XAI in COVID-19 Medical Imaging Informatics

In this section, we summarize the existing literature on AI-based clinical decision support systems for COVID-19 and examine the use of XAI methods. Specifically, we focus on the utilization of various XAI techniques, such as GradCAM[1], LIME[2], saliency analysis[3], CAM[4], attention mechanism[5], guided back-propagation[6], guided GradCAM[1], and others to highlight the features that are deemed important in the decision-making process of AI models. Some studies also incorporate expert validation to confirm the findings of XAI. This brief review aims to provide an overview of the current state of XAI in COVID-19 research and highlights the need for further evaluation of the interpretability of XAI in AI-leveraged clinical studies. A summary of related works of XAI generation, representation, and evaluation for COVID-19 radiographic imaging is available in Table S1 and in the following Section 4.

Table S1. Related Works of XAI in COVID-19 Radiographic Imaging

Reference	Data	XAI Generation	XAI Representation	XAI Evaluation
Karim, et al.[7]	CXR	Grad-CAM, Grad-CAM++, Layer-wise relevance propagation	Heatmap	N/A
Hou, et al.[8]	CXR	Saliency analysis	Heatmap	N/A
Ahsan, et al.[9]	CXR & CT	LIME	Heatmap & Feature importance	N/A
Malhotra, et al.[10]	CXR	Grad-CAM	Heatmap	N/A
Brunese, et al.[11]	CXR	Grad-CAM	Heatmap	Expert validation
Lee, et al.[12]	CXR	Grad-CAM	Heatmap	N/A
Mondal, et al.[13]	CXR & CT	Saliency analysis	Heatmap	Expert validation
Khan, et al.[14]	CXR	Grad-CAM	Heatmap	N/A
Ahsan, et al.[15]	CXR & CT	Saliency analysis, LIME	Heatmap & Feature importance	N/A
Zhang, et al.[16]	CXR	Saliency analysis, Grad-CAM	Heatmap	N/A
Teixeira, et al.[17]	CXR	LIME, Grad-CAM	Heatmap & Feature importance	N/A
Sharma, et al.[18]	CXR	Grad-CAM	Heatmap	N/A
Hu, et al.[19]	CXR	CAM	Heatmap	N/A
Ahsan, et al.[15]	CXR & CT	LIME	Feature importance	N/A
Wang, et al.[20]	CXR	GSInquire	Heatmap	N/A
Wang, et al.[21]	CXR	RIEC: Radiology reports	Semantic explanation	Expert validation
Aviles-Rivero, et al.[22]	CXR	Grad-CAM	Heatmap	N/A
Shi, et al.[23]	CXR & CT	Attention mechanism	Heatmap	N/A
Signoroni, et al.[24]	CXR	LIME	Heatmap	Expert validation
Chetoui, et al.[25]	CXR	Attention mechanism	Heatmap	N/A
Singh, et al.[26]	CXR	Grad-CAM	Heatmap	N/A
Zhang, et al.[27]	CXR & CT	Grad-CAM	Heatmap	N/A
Shi, et al.[28]	CXR & CT	Attention mechanism	Heatmap	N/A
Minaee, et al.[29]	CXR	Saliency analysis	Heatmap	N/A
Blain, et al.[30]	CXR	Saliency analysis	Heatmap	N/A

Table S1 continued from previous page

Reference	Data	XAI Generation	XAI Representation	XAI Evaluation
Alom, et al.[31]	CT	Saliency analysis	Heatmap	N/A
Arias-Garazón, et al.[32]	CXR	Saliency analysis	Heatmap	N/A
Sitaula, et al.[33]	CXR	Attention mechanism	Heatmap	N/A
Punn, et al.[34]	CXR	LIME	Feature importance	N/A
Duran-Lopez, et al.[35]	CXR	CAM	Heatmap	Expert validation
Li, et al.[36]	CXR & CT	Attention mechanism	Heatmap	N/A
Chatterjee, et al.[37]	CXR	Occlusion, Saliency analysis, Integrated gradients, Guided backpropagation, DeepLift, Neuron activation profiles	Heatmap	N/A
Karthik, et al.[38]	CXR	Saliency analysis, Guided back-propagation, Grad-CAM	Heatmap	N/A
Islam, et al.[39]	CXR	Grad-CAM	Heatmap	N/A
Gupta, et al.[40]	CXR	Grad-CAM	Heatmap	N/A
Mahmud, et al.[41]	CXR	Saliency analysis	Heatmap	Expert validation
Jain, et al.[42]	CXR	Grad-CAM	Heatmap	N/A
Ghoshal, et al.[43]	CXR	CAM, Guided backpropagation, Guided Gradient CAM	Heatmap	N/A
Lin et al.[44]	CXR	Guided Grad-CAM, Grad-CAM, Saliency analysis	Heatmap	N/A
Khakzar, et al.[45]	CXR	Inverse Bottleneck for Attribution (IBA), Regression IBA, Multi-Layer IBA	Feature importance	N/A
Panwar, et al.[46]	CXR & CT	Grad-CAM	Heatmap	N/A
Moujahid, et al.[47]	CXR	Grad-CAM	Heatmap	N/A
Basu, et al.[48]	CXR	Grad-CAM	Heatmap	N/A
Li, et al.[49]	CXR	Grad-CAM	Heatmap	N/A
Zhao, et al.[50]	CXR	Grad-CAM	Heatmap	N/A
Ozturk, et al.[51]	CXR	Grad-CAM	Heatmap	Expert validation
Karakanis, et al.[52]	CXR	Grad-CAM	Heatmap	N/A
Chowdhury, et al.[53]	CXR	Grad-CAM, Grad-CAM++	Heatmap	N/A
Nasiri, et al.[54]	CXR	Grad-CAM	Heatmap	N/A
Zhang, et al.[55]	CXR	Grad-CAM	Heatmap	N/A
Haghanifar, et al.[56]	CXR	Grad-CAM, LIME	Heatmap & Feature importance	Expert validation
Sadre, et al.[57]	CXR	Grad-CAM	Heatmap	N/A
Oh, et al.[58]	CXR	Probabilistic Grad-CAM	Heatmap	N/A
Wang, et al.[59]	CXR	Grad-CAM	Heatmap	N/A
Jin, et al.[60]	CT	Guided Grad-CAM	Heatmap	N/A

2 Method Details

2.1 Model Architecture

Table S2 outlines the baseline VGG-16 model architecture in this study.

Table S2. Baseline VGG-16 Model Parameter Details

Layer (type)	Input Shape	Param #	Tr. Param #
Conv2d-1	32, 3, 224, 224	1,792	1,792
ReLU-2	32, 64, 224, 224	0	0
Conv2d-3	32, 64, 224, 224	36,928	36,928
ReLU-4	32, 64, 224, 224	0	0
MaxPool2d-5	32, 64, 224, 224	0	0
Conv2d-6	32, 64, 112, 112	73,856	73,856
ReLU-7	32, 128, 112, 112	0	0
Conv2d-8	32, 128, 112, 112	147,584	147,584
ReLU-9	32, 128, 112, 112	0	0
MaxPool2d-10	32, 128, 112, 112	0	0
Conv2d-11	32, 128, 56, 56	295,168	295,168
ReLU-12	32, 256, 56, 56	0	0
Conv2d-13	32, 256, 56, 56	590,080	590,080
ReLU-14	32, 256, 56, 56	0	0
Conv2d-15	32, 256, 56, 56	590,080	590,080
ReLU-16	32, 256, 56, 56	0	0
MaxPool2d-17	32, 256, 56, 56	0	0
Conv2d-18	32, 256, 28, 28	1,180,160	1,180,160
ReLU-19	32, 512, 28, 28	0	0
Conv2d-20	32, 512, 28, 28	2,359,808	2,359,808
ReLU-21	32, 512, 28, 28	0	0
Conv2d-22	32, 512, 28, 28	2,359,808	2,359,808
ReLU-23	32, 512, 28, 28	0	0
MaxPool2d-24	32, 512, 28, 28	0	0
Conv2d-25	32, 512, 14, 14	2,359,808	2,359,808
ReLU-26	32, 512, 14, 14	0	0
Conv2d-27	32, 512, 14, 14	2,359,808	2,359,808
ReLU-28	32, 512, 14, 14	0	0
Conv2d-29	32, 512, 14, 14	2,359,808	2,359,808
ReLU-30	32, 512, 14, 14	0	0
MaxPool2d-31	32, 512, 14, 14	0	0
AdaptiveAvgPool2d-32	32, 512, 7, 7	0	0
Linear-33	32, 25088	102,764,544	102,764,544
ReLU-34	32, 4096	0	0
Dropout-35	32, 4096	0	0
Linear-36	32, 4096	16,781,312	16,781,312
ReLU-37	32, 4096	0	0
Dropout-38	32, 4096	0	0
Linear-39	32, 4096	4,097,000	4,097,000
Total params: 138,357,544			
Trainable params: 138,357,544			
Non-trainable params: 0			

2.2 Explanation Generation and Representation Details

For a given poisoned test image $\tilde{x}_i^{\text{test}}$ and the poisoned model f', we generate a saliency map s_i using an XAI method. In our experiments, we evaluate four gradient-based XAI methods using our proposed evaluation pipeline, including backpropagation[3], guided backpropagation[6], GradCAM[1], and guided GradCAM[1]. Additionally, we examine three perturbation-based XAI

methods, such as occlusion sensitivity[61], ablation study[62], and LIME[2]. In this section, we explain the detailed methodology of each XAI method as follows.

2.2.1 Backpropagation

Backpropagation[3] is a gradient-based method for computing the gradients of a target class concerning the input image in a neural network. More formally, given a model f', an input image $\tilde{x}_i^{\text{test}}$, and a target class y, backpropagation to generate a saliency map by pass the input image $\tilde{x}_i^{\text{test}}$ through the model to obtain the final activations A. We can then calculate the gradients of the target class y with respect to the input image $\tilde{x}_i^{\text{test}}$ using the chain rule:

$$\frac{\partial y}{\partial \tilde{x}_i^{\text{test}}} = \frac{\partial y}{\partial A} \cdot \frac{\partial A}{\partial \tilde{x}_i^{\text{test}}}.$$

The resulting gradient $\frac{\partial y}{\partial \tilde{x}_i^{\text{test}}}$ is then represented as a saliency map s_i that highlights the regions of the input image that are most important for the model's prediction.

2.2.2 Guided Backpropagation

Guided backpropagation[6] is a variation of the backpropagation method that uses guided activation to preserve positive activations while suppressing negative activations. Guided backpropagation prevents backward flow of negative gradients, thereby decreasing activation of higher layer unit.

2.2.3 GradCAM

GradCAM[1] calculates the gradient of a target concept, such as a class in a classification task, with respect to the activations of the final convolutional layer. The resulting visualization map highlights the important regions for predicting the concept. The gradient of the target class y with respect to the feature map activation A^k is calculated as $\frac{\partial y^c}{\partial A^k}$. Global average pooling is then applied to the gradients over the width i and height j dimensions to obtain the neuron importance weights α_k^c, which capture the importance of feature map k for the target class c:

$$\alpha_k^c = \frac{1}{Z} \sum_i \sum_j \frac{\partial y^c}{\partial A_{ij}^k}$$

A weighted combination of the forward activation maps is then obtained by summing the product of the neuron importance weights and the activation maps, followed by a ReLU activation:

$$L_{Grad-CAM}^c = \text{ReLU}\left(\sum_k \alpha_k^c A^k\right)$$

This produces a coarse heatmap highlighting the regions of the input image that are most important for predicting the target class.

2.2.4 Guided GradCAM

Guided Grad-CAM, also introduced in[1], improves the GradCAM method by using guided backpropagation. This enhances the resulting saliency map by preserving positive activations and suppressing negative ones. Guided backpropagation modifies gradients during backpropagation to flow only through positive activations. Guided Grad-CAM combines both guided backpropagation and Grad-CAM by element-wise multiplication. This results in a high-resolution, class-discriminative saliency map highlighting important features and their corresponding classes.

2.2.5 Occlusion Sensitivity

Occlusion sensitivity[61] generates a saliency map by occluding (i.e., hiding) different regions of the input image and observing the effect on the model's prediction. We first divide the input image $\tilde{x}_i^{\text{test}}$ into small square regions (i.e., occlusion windows). For each occlusion window, we then replace its contents with a neutral value (e.g., the mean pixel value of the image) and obtain the prediction y' of the model f' on the occluded input. The magnitude of the difference between the prediction y' on the occluded input and the prediction y on the original input can be used as a saliency map to highlight the regions of the input image that are most important for the prediction. The magnitude of the difference between the predictions on the original and occluded inputs provides a measure of how important each region of the input image is for the prediction. The regions with higher values in the saliency map are considered to be more important for the prediction.

2.2.6 Ablation Study

Feature ablation proposed by Meyes et al.[62] involves removing or modifying the features of an input instance and observing the effect on the prediction. Let $x_{i,j}$ denote the j-th feature of the input image $\tilde{x}_i^{\text{test}}$. The saliency map is obtained by observing the change in the prediction of the target class y when the feature $x_{i,j}$ is removed. Specifically, we divide the input features into multiple groups, and each group is altered collectively. This process aims to assess the significance of each group by monitoring the impact on the output when the groups are perturbed. Therefore, the magnitude of the gradient with respect to each feature indicates the importance of the feature for the prediction.

2.2.7 LIME

LIME[2] provides model-agnostic local explanations by explaining the nonlinear decision boundaries via perturbing instances around a specific instance. Specifically, LIME perturbs instance around a certain input and weight and observes the impact of the output:

$$\xi(\tilde{\mathbf{x}}^{\text{test}}) = \underset{s \in S}{\operatorname{argmin}} \mathscr{L}\left(f', s, \pi_x\right) + \Omega(s),$$

where explanation of a model is defined as $s \in S$ and S indicates class of potentially interpretable models; $\Omega(s)$ is the complexity of the explanation g; π_x measures locality around $\tilde{\mathbf{x}}^{\text{test}}$, and $\mathscr{L}(f', s, \pi)$ measures "unfaithfulness" of explanation s when approximating f in locality defined by π_x. Specifically, LIME aims to minimize $\mathscr{L}(\cdot)$ and have $\Omega(s)$ low enough to be interpretable. Therefore, the coefficients of the linear model s_i can be used as a saliency map to explain the prediction of the original model f' for the instance $\tilde{x}_i^{\text{test}}$.

2.3 Evaluation Metrics Details

2.3.1 SSIM Index

We introduce the detailed SSIM index calculation as follows:

$$\text{SSIM}(s^{(k)}, s^{(l)}) = \frac{(2\mu_{s^{(k)}}\mu_{s^{(l)}} + c_1)(2\sigma_{s^{(k)},s^{(l)}} + c_2)}{(\mu_{s^{(k)}}^2 + \mu_{s^{(l)}}^2 + c_1)(\sigma_{s^{(k)}}^2 + \sigma_{s^{(l)}}^2 + c_2)},$$

where $\mu_{s^{(k)}}$ and $\mu_{s^{(l)}}$ are the pixel sample mean of $s^{(k)}$ and $s^{(l)}$, respectively; $\sigma_{s^{(k)}}^2$ and $\sigma_{s^{(l)}}^2$ are the variances of $s^{(k)}$ and $s^{(l)}$, respectively; $\sigma_{s^{(k)},s^{(l)}}$ is the covariance between $s^{(k)}$ and $s^{(l)}$; and $c_1 = (k_1 L)^2, c_2 = (k_2 L)^2$ two variables to stabilize the division with weak denominator, where L denotes the dynamic range of the pixel-values and $k_1 = 0.01$ and $k_2 = 0.03$ by default.

3 Experiments Details

3.1 Hyper-Parameter Tuning

For hyper-parameter tuning, we visualize the dynamic trigger patterns with the pixel-wise perturbation amount $\varepsilon \in \{0.1, 0.3, 0.5\}$ during adversarial training in Figure S1 and selected $\varepsilon = 0.3$ for all dynamic patterns generation.

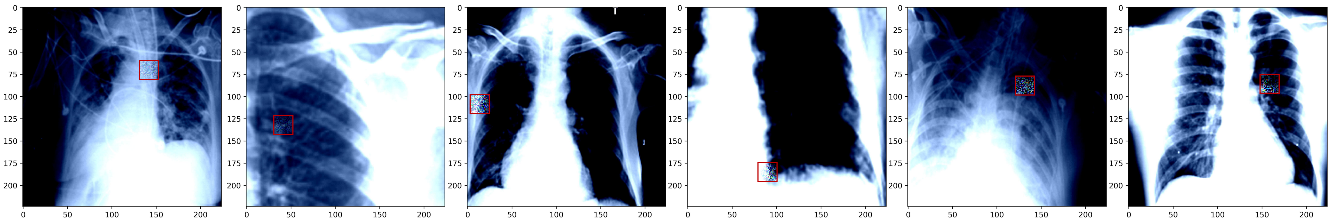

$(a)\ \epsilon = 0.1$ $(b)\ \epsilon = 0.3$ $(c)\ \epsilon = 0.5$

Figure S1. [double-column] For hyper-parameter tuning, we visualize the dynamic trigger patterns with the pixel-wise perturbation amount $\varepsilon \in \{0.1, 0.3, 0.5\}$ during adversarial training. We followed the settings from previous studies and set $\varepsilon = 0.3$ for dynamic pattern generation.

3.2 Baseline

Figure S2 shows the explainable results generated on baseline models without poisoning attack.

3.3 Attack Effectiveness (RQ1) Details

Table S3 shows the detailed trigger configuration settings with CDA and ASR results.

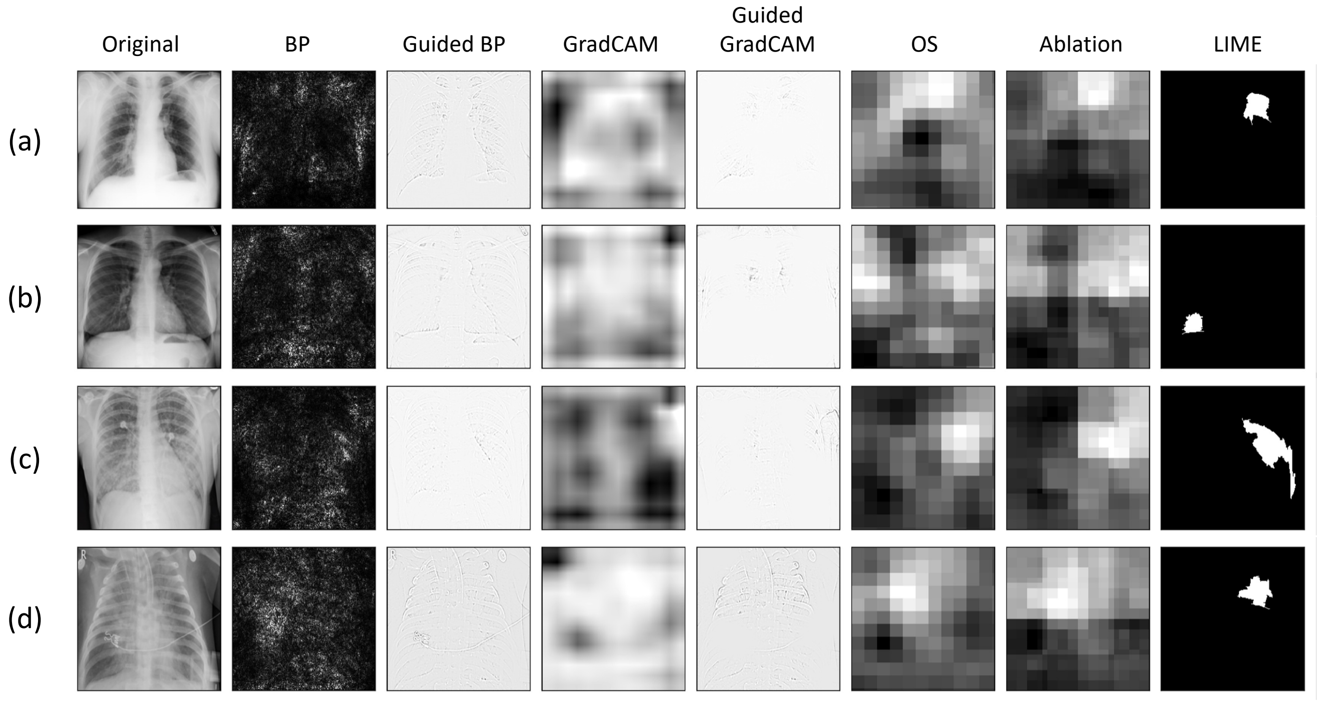

Figure S2. [double-column] Explainable results generated on baseline models without poisoning attack: (a) normal, (b) COVID-19, (c) lung opacity, (d) viral pneumonia.

Table S3. Trigger configuration and attack effectiveness performance.

ID	Trigger Type	Shape	Location	Size	CDA (avg.)	CDA (std.)	ASR (avg.)	ASR (std.)
1	static	square	corner	20*20	93.63	0.77	99.94	0.07
2	static	square	corner	40*40	92.86	1.52	99.99	0.01
3	static	square	corner	60*60	93.29	0.71	99.99	0.01
4	static	square	center	20*20	93.29	1.12	99.98	0.02
5	static	square	random	20*20	93.59	0.58	98.81	2.35
6	static	circle	corner	20*20	93.73	0.43	99.94	0.07
7	static	circle	center	20*20	93.75	0.92	99.99	0.01
8	static	circle	random	20*20	94.26	0.31	99.80	0.16
9	dynamic	random	random	20*20	91.94	1.57	99.99	0.01
10	dynamic	random	random	40*40	93.78	1.67	99.97	0.03
11	dynamic	random	random	60*60	92.96	1.71	99.99	0.01

3.4 Detection Effectiveness: Static Stamping (RQ2) Details

Figure S3 shows the additional detection results generated using XAI models during a poisoning attack with circle triggers.

3.5 Explanation Generation Efficiency (RQ5) Details

Table S4 shows the detailed running time of saliency maps generation using each XAI model for all experiments with both static and dynamic triggers.

4 XAI in COVID-19 Medical Imaging Applications

In this section, we include a detailed review of the existing literature on AI-based clinical decision support systems for COVID-19 and examine the use of XAI methods in this context.

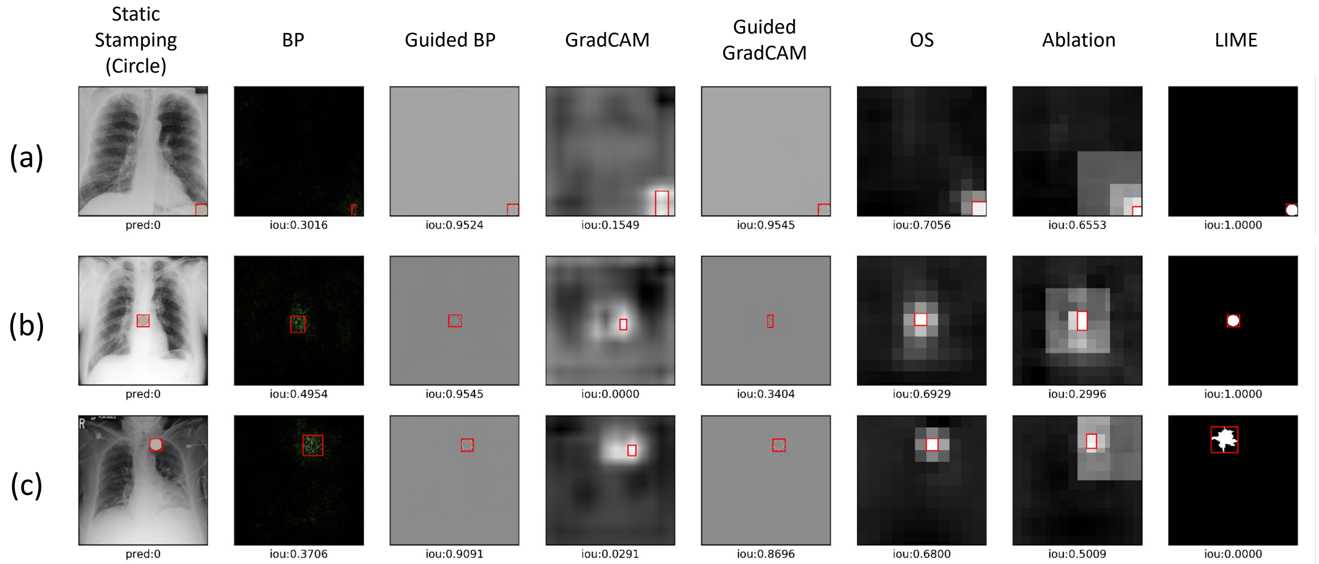

Figure S3. [double-column] Additional detection results generated using XAI models during a poisoning attack with static stamping: (a) Circle trigger in the corner, size 20×20; (b) Circle trigger at the center, size 20×20; (c) Circle trigger at a random location, size 20×20. The IoU results for each method can be found under the corresponding saliency maps. A higher IoU indicates better detection, as it signifies a larger overlap with the ground truth trigger.

Table S4. Running time (in seconds) of generating saliency maps using each XAI model for all 11 rounds of poisoning attack experiments with both static and dynamic triggers. The experimental ID is consistent with Table S3.

ID	BP	Guided BP	GradCAM	Guided GradCAM	OS	Ablation	LIME
1	0.57	0.62	**0.30**	0.79	17.71	39.52	21.88
2	0.76	0.78	**0.37**	1.01	5.14	50.97	24.82
3	0.95	0.96	**0.51**	1.24	2.50	54.06	22.53
4	0.81	0.84	**0.47**	1.14	21.05	43.86	22.27
5	0.72	0.75	**0.40**	1.05	24.73	54.50	22.17
6	0.82	0.88	**0.52**	1.20	20.43	42.78	22.32
7	0.57	0.61	**0.31**	0.76	13.99	28.79	23.18
8	0.76	0.81	**0.43**	1.16	19.35	38.64	21.69
9	0.33	0.35	**0.16**	0.52	11.09	25.05	21.12
10	0.58	0.60	**0.29**	0.89	5.04	53.02	31.51
11	0.22	0.23	**0.10**	0.33	0.65	19.11	20.61
avg.	0.64	0.68	**0.35**	0.92	12.88	40.94	23.10
std.	0.21	0.21	**0.13**	0.28	8.05	11.67	2.86

4.1 GradCAM

Karim et al.[7] proposed a Deepcovidexplainer to categorize COVID-19, pneumonia, and normal images using various COVID-19 Chest X-Ray (CXR) datasets. The model is an ensemble of VGG-19, ResNet-18, and DenseNet-161 architectures. To visualize the results, the authors employed visualization techniques such as GradCAM, GradCAM++, and layer-wise propagation, and provided human-interpretable explanations for the diagnosis.

Malhotra et al.[10] developed a deep learning model called COMiT-Net, which can classify and segment CXR images using COVID-19 CXR datasets from various sources. The model classified CXR images as normal, COVID-19, and others and also semantically segmented parts of the CXR images to increase explainability. The authors utilized GradCAM as a visualization method to demonstrate how COMiT-Net focused on regions of disease with a sensitivity of 96.89%.

Brunese et al.[11] proposed a method for discriminating between CXR images of healthy patients and those with different pulmonary diseases, including COVID-19, with the final step of utilizing an XAI method to provide explainability. This

three-step approach used transfer learning on VGG-16 and achieved an accuracy of 99%. To highlight COVID-19, GradCAM was utilized as an XAI method. Notably, the GradCAM visualizations were evaluated with markings provided by radiologists indicating where COVID-19 was present, and the model successfully highlighted the correct areas in most cases. The proposed approach provided a useful tool for the early detection of COVID-19 and other pulmonary diseases with expert validations.

Lee et al.[12] employed publicly available image data repositories to collect COVID-19 CXR images, and they also used the NIH CXR dataset to acquire normal and pneumonia datasets. Their model utilized VGG-16 and VGG-19 as backbone networks, achieving an AUROC of 0.95. GradCAM enabled the model to identify critical regions in CXR images, which helped in classifying the three categories.

Khan et al.[14] used deep transfer learning to train pretrained EfficientNet and VGG-16. Contrast enhancement to make enhanced images was followed by training of the model. Features were extracted through an improved discriminant canonical correlation analysis-based method along with Whale-Elephant Hearding optimization algorithm. Extreme learning machine was the final step in classifying final features. GradCAM visualization was used to highlight potential infectious or important regions in CXR images.

Zhang et al.[16] developed a model named CXRNet, which is an Encoder-Decoder-Encoder based architecture, to classify CXR images into three classes: healthy, bacterial pneumonia, and viral pneumonia. In order to provide visual explanations for model predictions, the authors compared the performance of three different XAI methods including GradCAM. The results showed that the proposed method was able to generate pixel-level visualizations with sharper details compared to the other methods.

Teixeira et al.[17] proposed a two-step classification system for COVID-19 detection in CXR images. The first step involved lung segmentation using a U-Net CNN architecture, while the second step utilized CNN models such as VGG, ResNet, and Inception for classification into three classes: lung opacity, COVID-19, and normal. To provide visual explanations for the classification results, the authors employed two XAI techniques, LIME and GradCAM. The results showed that for some of the classification models, the prediction for the lung opacity or normal class did not solely rely on the lung area, as evidenced by the XAI visualizations.

Sharma et al.[18] developed 16 deep learning models consisting of two segmentation networks and eight classification models to classify CXR images into four classes: Viral pneumonia, Bacterial pneumonia, Tuberculosis, and normal. The authors identified UNet as the best segmentation model, and UNet with Xception as the best segmentation-based classification model. To provide insight into the classification process, the authors utilized GradCAM visualization to identify the parts of the image that influenced the model's correct or incorrect classification. The results showed that the model made correct predictions by focusing on the upper parts of the lung images that were infected or had lesions after COVID-19 infection.

Aviles-Rivero et al.[22] developed a model named GraphXCovid, which utilized the COVIDx dataset for the classification of CXR images into three classes: healthy, pneumonia, and COVID-19. The authors proposed an optimization approach that involved using graph diffusion to establish a relationship between a small, labeled set of data and a more extensive, unlabeled set of data. The authors also employed GradCAM visualization to show the model's attention on the lung areas, which can be used as a user-friendly interface for radiologists.

Singh et al.[26] developed a deep learning framework called COVIDScreen, which incorporated image processing techniques, a segmentation model, and a modified stacked ensemble model for the classification of CXR images into COVID-19, normal, and pneumonia classes. The authors reported an accuracy of 98.67% for the proposed COVIDScreen model, which was created using four base Convolutional Neural Network (CNN) models and visualized using GradCAM for interpretability.

Zhang et al.[27] proposed a Multiple-Input Deep Convolutional Attention Network (MIDCAN) with a Convolutional Block Attention Module (CBAM) for the classification of CXR and CT scans into COVID-19 and healthy classes. The proposed model achieved a sensitivity of 98.1%, a specificity of 97.95%, and an accuracy of 98.02%. The authors also used GradCAM to demonstrate that the proposed model accurately captured lesions in both CT and CXR images. The results also showed that the inclusion of CBAM improved the model's performance.

Karthik et al.[38] developed a custom CNN architecture with unique convolutional filter learning patterns for different types of pneumonia or COVID-19. The proposed model classified CXR images into healthy, COVID-19, Bacterial pneumonia, and Viral pneumonia classes. Class saliency maps, guided backpropagation, and GradCAM were employed to visualize predictions.

Islam et al.[39] utilized a combined CNN and Long Short-Term Memory (LSTM) architecture for COVID-19 diagnosis on CXR images. The proposed model utilized CNN in conjunction with LSTM to classify CXR images into COVID-19, normal, and pneumonia classes. CNN was used for deep feature extraction, while detection was performed via LSTM. The authors utilized GradCAM to visualize the highlights of model prediction.

Gupta et al.[40] proposed InstaCovNet-19, a deep-learning model to identify COVID-19, pneumonia, and normal classes in CXR images. The proposed model uses pre-trained models as the base model for detecting COVID-19 efficiently. The authors used GradCAM to visualize the attention map of the proposed model, which showed that it focused on lung opacity, an indication of COVID-19 and pneumonia.

Jain et al.[42] developed a four-stage method to detect COVID-19 in CXR images. The method involved data augmentation, preprocessing stage, and a two-level deep network to classify normal, Bacterial pneumonia, Viral pneumonia, and COVID-19 classes. The authors used GradCAM visualization to show discriminatory features of CXR images.

Panwar et al.[46] proposed a deep transfer learning algorithm to classify CXR and CT scans into COVID-19, pneumonia, and non-COVID-19 that may have other pulmonary infections, and normal classes. The authors demonstrated that the proposed model can detect COVID-19 cases faster than RT-PCR tests. They used the GradCAM technique to provide a better understanding of the models.

Moujahid et al.[47] proposed a CNN-based method for classifying CXR images into three categories: COVID-19, other pneumonia, and normal. The authors utilized transfer learning by modifying the layers of three pre-trained models (VGG-16, VGG-19, and MobileNetV2) to improve the base models' performance. The results showed that the tuned VGG-19 model outperformed the others. The authors used GradCAM visualization to validate the accuracy of the predicted regions in the lungs.

Basu et al.[48] introduced the concept of domain extension transfer learning (DETL) to classify CXR images into four categories: normal, other disease, pneumonia, and COVID-19. The authors first trained AlexNet, VGGNet, and ResNet models to differentiate between diseased and normal CXR images, followed by a fine-tuning step for the final four classes. The authors demonstrated that the proposed model focused on ground glass opacity, which is clinically observed in COVID-induced pneumonia. GradCAM visualization was used to evaluate the accuracy of the predicted ground glass opacity.

Li et al.[49] proposed a Depthwise separable Convolutional Neural Network (DCNN) with LeNet-5, VGG-16, and ResNet-18 as base models. Additionally, the authors developed and tested a Dilated and Depthwise separable Convolutional Neural Network (DDCNN) with VGG-16 and ResNet-18 as base models. The authors proposed three methods of DCNNC and two novel methods of DDCNNC to classify CXR images into COVID-19, normal, and pneumonia. The study demonstrated that the DDCNNC-I had the highest accuracy. GradCAM visualization was used to analyze which proposed method contributed to the differences in the GradCAM visualizations.

In their recent study, Zhao et al.[50] investigated the impact of various parameters on the training of a CNN model for COVID-19 detection in CXR images. The authors employed the ResNet-50 X 1 architecture with a vanilla ResNet-v2 framework to classify CXR images into either SARS-CoV-2 negative or positive. The effectiveness of the proposed model was evaluated using GradCAM visualization, which indicated that the model could accurately localize SARS-COV-2 positive images. However, the model exhibited a tendency to focus on edges when classifying SARS-CoV-2 negative images.

Ozturk et al.[51] developed the DarkCovidNet model for binary classification of CXR images into COVID-19 and no-Findings, as well as multi-class classification into COVID-19, no-Findings, and pneumonia. The study showed high accuracy of 98.08% and 87.02% for binary and multi-class classification, respectively. The authors further evaluated the model's decisions using GradCAM visualization, which was then reviewed by a radiologist.

Karakanis et al.[52] proposed a method to address the limited availability of medical image data by employing a conditional generative adversarial network to generate synthetic images. They also developed a binary classification model to identify COVID-19 from normal images and a three-class classification model to classify COVID-19, normal, and pneumonia in CXR images. The results showed that the synthetic data improved the performance of CNN models in cases of limited data availability. Furthermore, the GradCAM visualizations revealed that the proposed binary classification model focused on the area around the lungs and the multi-classification model focused on specific areas of the thorax to make decisions.

Chowdhury et al.[53] developed Parallel-Dilated COVIDNet (PDCOVIDNet) to detect COVID-19 from CXR images. PDCOVIDNet comprises three components: feature extraction, detection, and visualization. The proposed model was trained to classify CXR images into three categories: COVID-19, normal, and viral pneumonia. GradCAM and GradCAM++ were utilized to visualize significant areas in the deep learning model's decision-making process. The study conducted a visualization analysis for each predicted class. GradCAM and GradCAM++ revealed comparable overlapping regions for COVID-19 and viral pneumonia classes, but both techniques failed to identify areas inside the lung.

In their recent study, Nasiri et al.[54] presented a method for detecting COVID-19 in CXR images using DenseNet169 and the Extreme Gradient Boosting algorithm (XGBoost). The method involved extracting features using DenseNet169, which were then used as input for XGBoost to classify images into COVID-19, pneumonia, and no-findings classes. The authors demonstrated that the developed method was successful in identifying relevant features and that the model focused on the lung area for making decisions, as revealed by the GradCAM method.

Zhang et al.[55] proposed a deep anomaly detection model to distinguish COVID-19 from non-COVID-19 CXR images. The model comprised three main components: a backbone network, a classification module, and an anomaly detection module. The study showed a sensitivity of 96.00% for detecting COVID-19 cases and a specificity of 70.65% for non-COVID-19 cases. The model's decision-making process was visualized using GradCAM, which revealed that the proposed method focused on regions within the lungs.

Haghanifar et al.[56] presented a new deep learning model, COVID-CXNet, for detecting COVID-19 in CXR images. A

publicly available dataset was collected from multiple sources to provide a large and diverse dataset. The CheXNet model was adopted for transfer learning to develop the COVID-CXNet model. The proposed model conducted a three-class classification of CXR images into COVID-19, normal, and community-acquired pneumonia (CAP) categories, with DenseNet-121 employed as a backbone model. The study employed Local Interpretable Model-Agnostic Explanations (LIME) to examine the contribution of the network architecture to the COVID-CXNet model's performance. LIME results indicated that the COVID-CXNet with the ROI-segmentation block achieved high performance when localizing pneumonia features, and that deeper CNN models were necessary to obtain high classification scores. GradCAM was utilized to depict the areas of CXR images used by the COVID-CXNet model to make its predictions, and showed that the ROI-segmentation block effectively identified regions of pneumonia.

Sadre et al.[57] proposed a novel protocol, called ROI Hide-and-Seek protocol, to investigate the effect of hiding or highlighting lung segmentation in CXR images on the performance of deep learning models. In this study, U-net was used to detect lungs to create five different representations of CXR images, which were used to train and test five deep learning architectures: COVID-Net CXR4 A, COVID-Net CXR3 A, AlexNet, VGG-11, and ResNet-50. GradCAM was employed to generate visualizations at the end of the proposed protocol to show highlighted regions in the CXR images. The visualization results indicated that the lung regions were usually highlighted in the images where the lungs were visible. In contrast, when the lungs were removed, GradCAM demonstrated localization to other regions such as the stomach and arms.

Oh et al.[58] proposed a novel patch-based convolutional neural network for the classification of CXR images into five classes: normal, tuberculosis, bacterial pneumonia, viral pneumonia, and COVID-19 pneumonia. The proposed model used random patch cropping to learn robust and diverse features from CXR images. The final decision of the model was made using majority voting. To address the lack of specificity of GradCAM, Probabilistic GradCAM was employed. The visualization results of the proposed model were consistent with clinical experts and showed considerable localization only in the COVID-19 class.

Wang et al.[59] presented a two-step framework for detecting COVID-19 in CXR images, which consisted of Discrimination-Deep Learning (DL) and Localization-DL. The Discrimination-DL classified CXR images into three classes: COVID-19, healthy, and CAP, while the Localization-DL classified COVID-19 images into the left, right lung, and bipulmonary classes. The study demonstrated a high accuracy of 98.71% for Discrimination-DL and 93.03% for Localization-DL. The GradCAM visualization revealed that the proposed model accurately localized the regions with abnormalities.

4.2 LIME

A deep learning model was developed by Ahsan et al.[9] to detect COVID-19 on both CT scans and CXR images. The study trained and tested eight deep learning networks, namely VGG-16, InceptionResNetV2, ResNet50, DenseNet201, VGG-19, MobilenetV2, NasNetMobile, and ResNet15V2, and showed that NasNetMobile achieved the highest accuracy on both types of images. Heatmap was used to visualize the activity of different layers, while LIME was used to provide interpretability to the model's results by highlighting top features that helped the model make decisions.

Ahsan et al.[15] implemented six deep CNN models, namely VGG-16, MobileNetV2, InceptionResNetV2, ResNet50, ResNet101, and VGG-19, to classify COVID-19 patients in a mixed dataset of CT scans and CXR images. The study demonstrated that MobileNetV2 achieved a high performance of about 95% accuracy on the mixed dataset. LIME was used to create superpixels, or the results of image over-segmentation, and showed the top features that led to the identification of COVID-19 classes.

Signoroni et al.[24] proposed a method called BS-Net that assesses the severity of COVID-19 by computing six Brixia scores on CXR images. The method includes segmentation, alignment, and score prediction and is adaptable to different modalities, acquisition directions, and patient conditions. The study employed weakly supervised learning and developed a new method for generating explainable maps that highlights important regions for producing a specific score, similar to LIME. The explainable maps produced clear visualizations of the regions that contribute to a specific score.

Punn et al.[34] applied transfer learning to ResNet, Inception-v3, Inception ResNet-v2, DenseNet169, and NASNetLarge to identify COVID-19 cases from CXR images. The study used binary (normal and COVID-19) and multi-class (COVID-19, pneumonia, and normal) classification. The NASNetLarge model showed the best performance compared to other models. The study utilized LIME and CAM to identify salient regions contributing to classification results.

4.3 Saliency Analysis

Mondal et al.[13] proposed an Explainable Vision Transformer-based COVID-19 Screening Using Radiography (xViTCOS) to classify CT scans and CXR images into normal, pneumonia, and COVID-19 classes. Vision transformers were used instead of CNN. To overcome the problem of data scarcity, multi-stage transfer learning was employed. The Gradient Attention Rollout algorithm was utilized to highlight meaningful regions contributing to classification results, and radiologists validated the explainability of the model.

Minaee et al.[29] trained four popular CNNs, namely ResNet18, ResNet50, SqueezeNet, and DenseNet-121, to identify

COVID-19, non-COVID-19, and other diseases from CXR images. The study demonstrated a sensitivity of 98% and a specificity of 90%. Heatmaps were generated to visualize the important regions of the images and showed high consistency with the regions determined by radiologists.

Blain et al.[30] developed a deep-learning model that conducts lung segmentation and detects Interstitial Opacity and Alveolar Opacity from CXR images. The lung segmentation model used a modified U-Net, while DenseNet121 was used for the image classification network. The study also investigated clinical factors such as age and symptoms to determine the severity of opacity. Heatmaps were generated to visualize the decision-making process of the model.

Alom et al.[31] employed a combination of CT and CXR images for the purpose of distinguishing COVID-19 cases from normal ones. The authors utilized transfer learning via Inception Residual Recurrent CNN for classification and the NABLA-N model network was employed for segmenting infected regions. The study reported an accuracy of 98.78% for CT scans and 84.67% for CXR images. In addition, heatmaps were utilized to visualize the model's outcomes, indicating both instances of accurate and erroneous detection.

Arias-Garzón et al.[32] utilized VGG-19 and U-Net to classify CXR images into COVID-19 positive and negative classes. The proposed scheme consisted of three steps: lung segmentation to remove irrelevant surroundings, a transfer learning-based classification model, and result analysis and visualization. The proposed model achieved an accuracy of approximately 97%. Heatmaps were generated to identify the regions of the image that were crucial for decision-making.

Mahmud et al.[41] introduced CovXNet, a CNN that employed depthwise dilated convolutions for classifying CXR images into four categories: normal, Viral pneumonia, Bacterial pneumonia, and COVID-19. A stacking algorithm was used to integrate extracted features from different image resolutions. Heatmaps were superimposed with CXR images to visualize localizations, and this visualization method was analyzed for various clinical features of the image.

4.4 CAM

Hu et al.[19] introduced the Multi-Input Transfer Learning COVID-Net fuzzy CNN to classify CXR images into COVID-19 or normal classes. VGG-16, ResNet, InceptionV3, and EfficientNets were used as base models. The authors used CAM to demonstrate that the localization was more confined with fuzzy filters compared to models that were not trained with fuzzy filters.

Duran-Lopez et al.[35] proposed a framework called COVID-XNet to classify CXR images into COVID-19 and normal classes. The COVID-XNet framework included preprocessing algorithms and CNN for classification. The model achieved an accuracy of 94.43% and an AUROC of 0.988. CAM was utilized to identify salient regions in the datasets with ground truth descriptions, and the results were validated by radiologists.

4.5 Attention Mechanism

Shi et al.[23] proposed Explainable Attention-based Model (EXAM) to classify CXR and CT scans into COVID-19, pneumonia, and normal classes, along with visual interpretation. The proposed model used Self-Adaptive Dense Block (SADB), Infection-Aware Attention Module (IAAM), and other layers to extract features. The attention maps generated by EXAM revealed that ground-glass opacity and consolidation opacity in pulmonary regions are important factors for diagnosis.

Chetoui et al.[25] used vision transformers (ViT) to detect COVID-19 in CXR images. The ViT models were fine-tuned and classified CXR images to normal, pneumonia, and COVID-19 classes. The study obtained 0.99 AUROC for multi-class classification and achieved 0.99 sensitivity for the COVID-19 class. The attention map located important parts for COVID-19 classification and highlighted opacity on the lungs for predicting pneumonia class.

Shi et al.[28] used both chest CT and CXR images to create an explainable classification model that classifies COVID-19, normal, and pneumonia. The proposed method based its structure on a knowledge distillation network, where attention transfer direction is handed over to the student network from the teacher network. The teacher network extracts features and focuses on infected regions, generating attention visualizations. An image fusion module combines the original input with attention information from the teacher network. Attention maps highlighted detailed parts of the input image.

Sitaula et al.[33] created a novel attention-based deep learning model to classify CXR images to COVID-19, pneumonia, and no-findings class. VGG-16 with an attention model was proposed, capturing the spatial relationship between the regions of interest (ROIs) in images. This approach required fewer parameters and could be trained end-to-end. The attention map was utilized as a visualization technique to highlight the defect areas in the upper regions of the lungs.

Li et al.[36] presented the Contrastive Multi-task Convolutional Neural Network (CMT-CNN) which performed two tasks: COVID-19 diagnosis and Contrastive Learning. The former conducts binary classification (COVID-19 and other conditions) and ternary classification (COVID-19, other pneumonia, and normal). The latter made predictions that were invariant to transformations. Attention maps revealed that CMT-CNN was capable of making finer localizations compared to a typical CNN, with frequent highlighting of the upper regions of the lungs.

4.6 Guided Backpropagation and Guided GradCAM

Chatterjee et al.[37] investigated the classification of CXR images into multiple labels for diseases and supertypes, and into multiple classes, such as healthy, Viral, Bacterial, and others. The study trained five deep learning models – ResNet18, ResNet34, InceptionV3, InceptionResNetV2, and DenseNet161 – along with their Ensembles. To gain interpretability of the model's results, seven explainable AI methods were used, including occlusion, saliency, input X gradient, guided backpropagation, integrated gradients, DeepLIFT, and neuron activation profiles. The visualizations showed that more complex models tended to be less interpretable, and vice versa. For example, DenseNet161 showed the highest quantitative performance but had the worst localization areas.

Ghoshal et al.[43] investigated the uncertainty of prediction in detecting COVID-19 and its relation to the accuracy of the prediction. The study fine-tuned a pre-trained ResNet50V2 to classify CXR images into normal, Bacterial, Viral, and COVID-19 classes. The study demonstrated that estimating uncertainty in deep learning models can produce more robust and reliable predictions. Guided Backpropagation, Guided GradCAM, CAM, and Gradients were used to understand the decision of the models.

Lin et al.[44] utilized public open datasets of CXR images to classify into four classes (COVID-19, normal, Bacterial, and Viral) and three classes (COVID-19, normal, and pneumonia). The proposed method had five steps: image preprocessing, CNN models, XAI visualization, meta classifier for the first CNN ensemble, and second ensemble for meta classifiers. Using GradCAM, it was shown that applying the model with ROI and mask process helps CNN focus on the lung area. Saliency map, Guided Backpropagation, and Guided GradCAM provided finer visualizations compared to GradCAMs.

Jin et al.[60] developed a system to classify CT scans into four classes: COVID-19, CAP, influenza, and non-pneumonia. The system consisted of five components: lung segmentation, slice diagnosis network, COVID-infected slice locating network, interpretable visualization, and image phenotype analysis. To understand the model's decision, Guided GradCAM was used to generate visualizations of highlighted regions. Additionally, t-SNE was utilized to visualize the latent space in a 2D plane, which allowed for a better understanding of the distribution of the features.

4.7 Other XAI Methods

Hou et al.[8] developed an explainable deep CNN that classifies CXR images to normal, COVID-19, and other virus infections. The proposed framework consists of two steps, where the first CNN detects normal, Infected by bacteria, and Infected by virus classes, and the second CNN takes in the class Infected by virus to classify into the final three classes. The study achieved an average accuracy of about 96%. The proposed model produced an explainable model which highlights important regions with confidence scores per class.

Wang et al.[20] presented COVID-Net, a deep CNN for the detection of COVID-19 from CXR images that predicts three classes: no infection, non-COVID-19 infection (viral and bacterial), and COVID-19 viral infection, achieving an accuracy of 93.3%. To add an interpretable qualitative analysis of COVID-Net, GSInquire was employed, which identified important features for decision-making along with visualizations. Interpretation by GSInquire revealed that COVID-Net used areas in the lungs in CXR images to detect COVID-19 infection.

Wang et al.[21] proposed a semantic-powered and explainable mechanism that uses radiology reports as interpretable information to classify CXR images into five classes: viral pneumonia, normal, COVID-19, SARS, and bacterial pneumonia. Pre-trained models extracted features from CXR images, and an LSTM extracted radiology report features. The Report Image Explanation Cell (RIEC) employed radiology reports as interpretable information to explain black-box deep learning models.

Khakzar et al.[45] proposed the Inverse, Regression, and Multi-layer Information Bottleneck Attribution (IBA) method. The classification model classified CXR images into eight pathologies and the regression model predicted the total severity score. Inverse, Regression, and Multi-layer IBA each produced visualizations that showed highlighted regions. Inverse IBA identified all regions with important information, Regression IBA showed informative regions for regression models, and Multi-layer IBA produced fine-grained visualizations.

References

1. Selvaraju, R. R. *et al.* Grad-cam: Visual explanations from deep networks via gradient-based localization. In *Proceedings of the IEEE international conference on computer vision*, 618–626 (2017).

2. Ribeiro, M. T., Singh, S. & Guestrin, C. " why should i trust you?" explaining the predictions of any classifier. In *Proceedings of the 22nd ACM SIGKDD international conference on knowledge discovery and data mining*, 1135–1144 (2016).

3. Simonyan, K., Vedaldi, A. & Zisserman, A. Deep inside convolutional networks: Visualising image classification models and saliency maps. *arXiv preprint arXiv:1312.6034* (2013).

4. Zhou, B., Khosla, A., Lapedriza, A., Oliva, A. & Torralba, A. Learning deep features for discriminative localization. In *Proceedings of the IEEE conference on computer vision and pattern recognition*, 2921–2929 (2016).

5. Wang, X., Girshick, R., Gupta, A. & He, K. Non-local neural networks. In *Proceedings of the IEEE conference on computer vision and pattern recognition*, 7794–7803 (2018).

6. Springenberg, J. T., Dosovitskiy, A., Brox, T. & Riedmiller, M. Striving for simplicity: The all convolutional net. *arXiv preprint arXiv:1412.6806* (2014).

7. Karim, M. R. *et al.* Deepcovidexplainer: explainable covid-19 diagnosis from chest x-ray images. In *2020 IEEE international conference on bioinformatics and biomedicine (BIBM)*, 1034–1037 (IEEE, 2020).

8. Giełczyk, A., Marciniak, A., Tarczewska, M. & Lutowski, Z. Pre-processing methods in chest x-ray image classification. *Plos one* **17**, e0265949 (2022).

9. Ahsan, M. M. *et al.* Study of different deep learning approach with explainable ai for screening patients with covid-19 symptoms: Using ct scan and chest x-ray image dataset. *arXiv preprint arXiv:2007.12525* (2020).

10. Malhotra, A. *et al.* Multi-task driven explainable diagnosis of covid-19 using chest x-ray images. *Pattern Recognit.* **122**, 108243 (2022).

11. Brunese, L., Mercaldo, F., Reginelli, A. & Santone, A. Explainable deep learning for pulmonary disease and coronavirus covid-19 detection from x-rays. *Comput. Methods Programs Biomed.* **196**, 105608 (2020).

12. Lee, K.-S. *et al.* Evaluation of scalability and degree of fine-tuning of deep convolutional neural networks for covid-19 screening on chest x-ray images using explainable deep-learning algorithm. *J. personalized medicine* **10**, 213 (2020).

13. Mondal, A. K., Bhattacharjee, A., Singla, P. & Prathosh, A. xvitcos: explainable vision transformer based covid-19 screening using radiography. *IEEE J. Transl. Eng. Heal. Medicine* **10**, 1–10 (2021).

14. Khan, M. A. *et al.* Covid-19 classification from chest x-ray images: a framework of deep explainable artificial intelligence. *Comput. Intell. Neurosci.* **2022** (2022).

15. Ahsan, M. M., Nazim, R., Siddique, Z. & Huebner, P. Detection of covid-19 patients from ct scan and chest x-ray data using modified mobilenetv2 and lime. In *Healthcare*, vol. 9, 1099 (MDPI, 2021).

16. Zhang, X. *et al.* Cxr-net: An encoder-decoder-encoder multitask deep neural network for explainable and accurate diagnosis of covid-19 pneumonia with chest x-ray images. *arXiv preprint arXiv:2110.10813* (2021).

17. Teixeira, L. O. *et al.* Impact of lung segmentation on the diagnosis and explanation of covid-19 in chest x-ray images. *Sensors* **21**, 7116 (2021).

18. Sharma, N. *et al.* Segmentation-based classification deep learning model embedded with explainable ai for covid-19 detection in chest x-ray scans. *Diagnostics* **12**, 2132 (2022).

19. Hu, Q. *et al.* Explainable artificial intelligence-based edge fuzzy images for covid-19 detection and identification. *Appl. Soft Comput.* **123**, 108966 (2022).

20. Wang, L., Lin, Z. Q. & Wong, A. Covid-net: A tailored deep convolutional neural network design for detection of covid-19 cases from chest x-ray images. *Sci. reports* **10**, 1–12 (2020).

21. Wang, Y. *et al.* Semantic-powered explainable model-free few-shot learning scheme of diagnosing covid-19 on chest x-ray. *IEEE J. Biomed. Heal. Informatics* **26**, 5870–5882 (2022).

22. Aviles-Rivero, A. I., Sellars, P., Schönlieb, C.-B. & Papadakis, N. Graphxcovid: explainable deep graph diffusion pseudo-labelling for identifying covid-19 on chest x-rays. *Pattern Recognit.* **122**, 108274 (2022).

23. Shi, W., Tong, L., Zhuang, Y., Zhu, Y. & Wang, M. D. Exam: an explainable attention-based model for covid-19 automatic diagnosis. In *Proceedings of the 11th ACM international conference on bioinformatics, computational biology and health informatics*, 1–6 (2020).

24. Signoroni, A. *et al.* Bs-net: Learning covid-19 pneumonia severity on a large chest x-ray dataset. *Med. Image Analysis* **71**, 102046 (2021).

25. Chetoui, M. & Akhloufi, M. A. Explainable vision transformers and radiomics for covid-19 detection in chest x-rays. *J. Clin. Medicine* **11**, 3013 (2022).

26. Singh, R. K., Pandey, R. & Babu, R. N. Covidscreen: explainable deep learning framework for differential diagnosis of covid-19 using chest x-rays. *Neural Comput. Appl.* **33**, 8871–8892 (2021).

27. Zhang, Y.-D., Zhang, Z., Zhang, X. & Wang, S.-H. Midcan: A multiple input deep convolutional attention network for covid-19 diagnosis based on chest ct and chest x-ray. *Pattern recognition letters* **150**, 8–16 (2021).

28. Shi, W., Tong, L., Zhu, Y. & Wang, M. D. Covid-19 automatic diagnosis with radiographic imaging: explainable attention transfer deep neural networks. *IEEE J. Biomed. Heal. Informatics* **25**, 2376–2387 (2021).

29. Minaee, S., Kafieh, R., Sonka, M., Yazdani, S. & Soufi, G. J. Deep-covid: Predicting covid-19 from chest x-ray images using deep transfer learning. *Med. image analysis* **65**, 101794 (2020).

30. Blain, M. *et al.* Determination of disease severity in covid-19 patients using deep learning in chest x-ray images. *Diagn. interventional radiology* **27**, 20 (2021).

31. Alom, M. Z., Rahman, M., Nasrin, M. S., Taha, T. M. & Asari, V. K. Covid_mtnet: Covid-19 detection with multi-task deep learning approaches. *arXiv preprint arXiv:2004.03747* (2020).

32. Arias-Garzón, D. *et al.* Covid-19 detection in x-ray images using convolutional neural networks. *Mach. Learn. with Appl.* **6**, 100138 (2021).

33. Sitaula, C. & Hossain, M. B. Attention-based vgg-16 model for covid-19 chest x-ray image classification. *Appl. Intell.* **51**, 2850–2863 (2021).

34. Punn, N. S. & Agarwal, S. Automated diagnosis of covid-19 with limited posteroanterior chest x-ray images using fine-tuned deep neural networks. *Appl. Intell.* **51**, 2689–2702 (2021).

35. Duran-Lopez, L., Dominguez-Morales, J. P., Corral-Jaime, J., Vicente-Diaz, S. & Linares-Barranco, A. Covid-xnet: A custom deep learning system to diagnose and locate covid-19 in chest x-ray images. *Appl. Sci.* **10**, 5683 (2020).

36. Li, J. *et al.* Multi-task contrastive learning for automatic ct and x-ray diagnosis of covid-19. *Pattern recognition* **114**, 107848 (2021).

37. Chatterjee, S. *et al.* Exploration of interpretability techniques for deep covid-19 classification using chest x-ray images. *arXiv preprint arXiv:2006.02570* (2020).

38. Karthik, R., Menaka, R. & Hariharan, M. Learning distinctive filters for covid-19 detection from chest x-ray using shuffled residual cnn. *Appl. Soft Comput.* **99**, 106744 (2021).

39. Islam, M. Z., Islam, M. M. & Asraf, A. A combined deep cnn-lstm network for the detection of novel coronavirus (covid-19) using x-ray images. *Informatics medicine unlocked* **20**, 100412 (2020).

40. Gupta, A., Gupta, S., Katarya, R. *et al.* Instacovnet-19: A deep learning classification model for the detection of covid-19 patients using chest x-ray. *Appl. Soft Comput.* **99**, 106859 (2021).

41. Mahmud, T., Rahman, M. A. & Fattah, S. A. Covxnet: A multi-dilation convolutional neural network for automatic covid-19 and other pneumonia detection from chest x-ray images with transferable multi-receptive feature optimization. *Comput. biology medicine* **122**, 103869 (2020).

42. Jain, G., Mittal, D., Thakur, D. & Mittal, M. K. A deep learning approach to detect covid-19 coronavirus with x-ray images. *Biocybern. biomedical engineering* **40**, 1391–1405 (2020).

43. Ghoshal, B. & Tucker, A. Estimating uncertainty and interpretability in deep learning for coronavirus (covid-19) detection. *arXiv preprint arXiv:2003.10769* (2020).

44. Lin, T.-C. & Lee, H.-C. Covid-19 chest radiography images analysis based on integration of image preprocess, guided grad-cam, machine learning and risk management. In *Proceedings of the 4th International Conference on Medical and Health Informatics*, 281–288 (2020).

45. Khakzar, A. *et al.* Explaining covid-19 and thoracic pathology model predictions by identifying informative input features. In *Medical Image Computing and Computer Assisted Intervention–MICCAI 2021: 24th International Conference, Strasbourg, France, September 27–October 1, 2021, Proceedings, Part III 24*, 391–401 (Springer, 2021).

46. Panwar, H. *et al.* A deep learning and grad-cam based color visualization approach for fast detection of covid-19 cases using chest x-ray and ct-scan images. *Chaos, Solitons & Fractals* **140**, 110190 (2020).

47. Moujahid, H. *et al.* Combining cnn and grad-cam for covid-19 disease prediction and visual explanation. *Intell. Autom. & Soft Comput.* **32** (2022).

48. Basu, S., Mitra, S. & Saha, N. Deep learning for screening covid-19 using chest x-ray images. In *2020 IEEE Symposium Series on Computational Intelligence (SSCI)*, 2521–2527 (IEEE, 2020).

49. Li, X., Zhai, M. & Sun, J. Ddcnnc: Dilated and depthwise separable convolutional neural network for diagnosis covid-19 via chest x-ray images. *Int. J. Cogn. Comput. Eng.* **2**, 71–82 (2021).

50. Zhao, W., Jiang, W. & Qiu, X. Fine-tuning convolutional neural networks for covid-19 detection from chest x-ray images. *Diagnostics* **11**, 1887 (2021).

51. Ozturk, T. *et al.* Automated detection of covid-19 cases using deep neural networks with x-ray images. *Comput. biology medicine* **121**, 103792 (2020).

52. Karakanis, S. & Leontidis, G. Lightweight deep learning models for detecting covid-19 from chest x-ray images. *Comput. biology medicine* **130**, 104181 (2021).

53. Chowdhury, N. K., Rahman, M. M. & Kabir, M. A. Pdcovidnet: a parallel-dilated convolutional neural network architecture for detecting covid-19 from chest x-ray images. *Heal. information science systems* **8**, 27 (2020).

54. Nasiri, H. & Hasani, S. Automated detection of covid-19 cases from chest x-ray images using deep neural network and xgboost. *Radiography* **28**, 732–738 (2022).

55. Zhang, J., Xie, Y., Li, Y., Shen, C. & Xia, Y. Covid-19 screening on chest x-ray images using deep learning based anomaly detection. *arXiv preprint arXiv:2003.12338* **27**, 141 (2020).

56. Haghanifar, A., Majdabadi, M. M., Choi, Y., Deivalakshmi, S. & Ko, S. Covid-cxnet: Detecting covid-19 in frontal chest x-ray images using deep learning. *Multimed. Tools Appl.* **81**, 30615–30645 (2022).

57. Sadre, R., Sundaram, B., Majumdar, S. & Ushizima, D. Validating deep learning inference during chest x-ray classification for covid-19 screening. *Sci. reports* **11**, 16075 (2021).

58. Oh, Y., Park, S. & Ye, J. C. Deep learning covid-19 features on cxr using limited training data sets. *IEEE transactions on medical imaging* **39**, 2688–2700 (2020).

59. Wang, Z. *et al.* Automatically discriminating and localizing covid-19 from community-acquired pneumonia on chest x-rays. *Pattern recognition* **110**, 107613 (2021).

60. Jin, C. *et al.* Development and evaluation of an artificial intelligence system for covid-19 diagnosis. *Nat. communications* **11**, 5088 (2020).

61. Zeiler, M. D. & Fergus, R. Visualizing and understanding convolutional networks. In *Computer Vision–ECCV 2014: 13th European Conference, Zurich, Switzerland, September 6-12, 2014, Proceedings, Part I 13*, 818–833 (Springer, 2014).

62. Meyes, R., Lu, M., de Puiseau, C. W. & Meisen, T. Ablation studies in artificial neural networks. *arXiv preprint arXiv:1901.08644* (2019).
